# Enhanced Stability and Properties of Benzene‐1,3,5‐Tricarboxamide Supramolecular Copolymers through Engineered Coupled Equilibria**

**DOI:** 10.1002/anie.202421991

**Published:** 2024-12-08

**Authors:** Huanjun Kong, Antonio Valverde‐González, Régina Maruchenko, Laurent Bouteiller, Matthieu Raynal

**Affiliations:** ^1^ Sorbonne Université CNRS Institut Parisien de Chimie Moléculaire 4 Place Jussieu 75005 Paris France

**Keywords:** benzene-1,3,5-tricarboxamide, competing assemblies, coupled equilibria, helical catalyst, supramolecular copolymerization

## Abstract

Improving the stability of multi‐component and functional assemblies such as supramolecular copolymers without impeding their dynamicity is key for their implementation as innovative materials. Up to now, this has been achieved by a trial‐and‐error approach, requiring the time‐consuming characterization of a series of supramolecular coassemblies. We report herein that this is possible to significantly enhance the stability of supramolecular copolymers by a minimal change in the chemical nature of one of the interacting monomers. This is achieved by replacing an ester function by an ether function in the structure of a chiral benzene‐1,3,5‐tricarboxamide (BTA) monomer, used as “sergeant”, coassembled with achiral monomers, the “soldiers”. Pseudo‐phase diagrams, constructed by probing the nature of the coassemblies with multifarious analytical techniques, confirm that the greater stability of the resulting copolymers is mainly due to the minimization of competing species. This leads to better rheological and catalytic properties of the corresponding supramolecular copolymers. Favouring coassembly over undesired assembly pathways must be considered as a blueprint for the development of better‐performing supramolecular multi‐component systems.

## Graphical abstract

Replacing an ester by an ether function in the side chain of a chiral benzene1,3,5‐tricarboxamide (BTA) monomer (magenta and red disks, resp.) greatly favours its copolymerization with achiral BTA monomers (green disk), due to the minimization of competing species. The resulting supramolecular copolymers exhibit greater stability which translates into better catalytic and rheological properties.

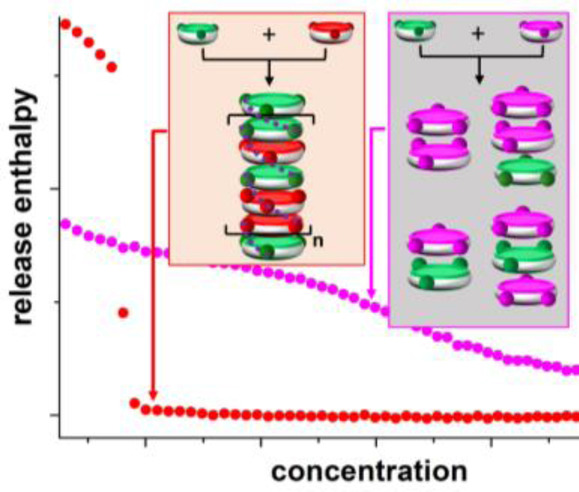

## Introduction

The assembly of various components, some of them embedding chiral and/or functional groups, constitutes a remarkably simple approach for the construction of tuneable and sophisticated supramolecular architectures for applications^[1]^ as optoelectronic,[^2^, ^3^, ^4^] biomedical,^[5]^ and catalytic materials.^[6]^ Great efforts have been devoted towards a better understanding of the parameters allowing to control the structure, stability and dynamicity of multi‐component and functional assemblies. Competing groups embedded in functional monomers could significantly impact the assembly route leading to supramolecular polymorphism,^[7]^ i.e. to different competing supramolecular structures under thermodynamic^[8]^ or kinetic control.^[9]^ “Pathway complexity”[^10^, ^11^] notably paves the way towards kinetically controlled polymerization that can be used for exquisite control of the structure, as shown by recent examples of multiblock supramolecular copolymers (SCPs).[^12^, ^13^] This structural control usually comes at the expense of the dynamicity and stability properties of the resulting SCPs.^[14]^ Controlling the properties of a SCP formed under thermodynamic equilibrium is usually done by tailoring the process leading to the assembly of the monomer(s).^[15]^ Molecular additives (or solvents) can then be added to mediate the assembly process through coupled equilibria between additive/monomer, additive/additive and monomer/monomer leading to unusual behaviors such as dilution‐induced or heating‐promoted polymerization,[^16^, ^17^, ^18^, ^19^] self‐correction,^[20]^ helicity inversion,^[21]^ and retardation of the polymerization process.[^22^, ^23^] When several functional monomers are mixed together, the assembly process is not trivial, and this is particularly true for SCP assembled through a cooperative mechanism. In that case, competing assembly pathways and the free energy of the heterointeraction between the monomers will dictate the composition, length, and microstructure of the resulting supramolecular copolymer.^[24]^

In this area, most examples in the literature deal with mixing two complementary monomers, with the influence of each other on the coassembly process being determined *a posteriori* by characterization of the SCP. A trial‐and‐error approach and/or chemist ingenuity in the design of the monomers led to examples in which one of the monomers helps solubilizing the SCP (“coformer” approach),[^25^, ^26^] or interacts efficiently with the other monomer to favour intercalation into the same SCP.[^27^, ^28^, ^29^, ^30^, ^31^, ^32^, ^33^, ^34^] Higher thermodynamic stability of SCP, i.e. their ability to maintain their integrity upon dilution and heating, is hard to achieve upon simple design of the monomer structure, probably because of the difficulty to properly engineer the aforementioned free energy of heterointeraction. Meijer and co‐workers reported the higher stability in water for an amphiphilic benzene‐1,3,5‐tricarboxamide (BTA) monomer when coassembled with a BTA monomer decorated with dendronized ethylene glycol‐based^[35]^ or monosaccharide side chains.^[5]^ Our group recently found that adding an achiral monomer significantly increases the stability of a two‐component BTA‐based SCP in toluene.[^36^, ^37^] In these examples, the enhanced stability was attributed to a subtle influence of the co‐monomer leading locally to a decrease in the number of chiral or structural defects.

**Scheme 1:**
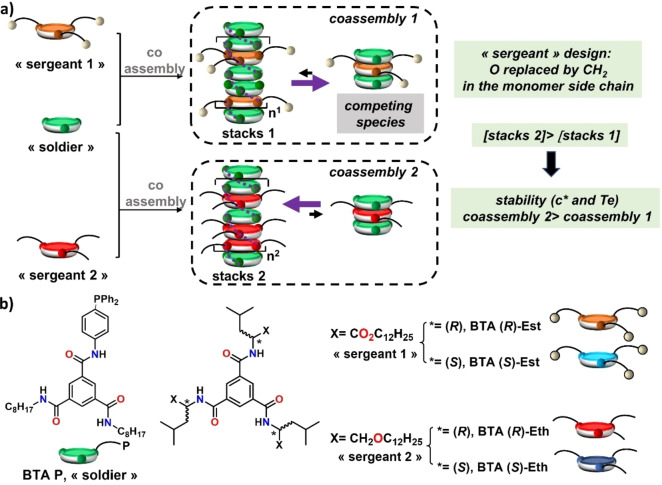
a) Coupled equilibria and their influence on the thermodynamic stability of supramolecular copolymers or coassemblies. An achiral monomer (“soldier”, green disk) is copolymerized with either “sergeant 1” (orange disk) or “sergeant 2” (red disk) that differs only by the nature of the lateral groups located in their side chains. The supramolecular helical copolymer formed by coassembly of the “soldier” and “sergeant 2” is more stable because competing species are minimized. For the sake of simplicity, only polymer (stacks, the desired SCP) and competing species are represented. b) Chemical structures of the main BTA monomers investigated in this study.

In an unfavourable but frequently met scenario, the co‐monomer exhibits a preferred interaction with the monomer leading to its sequestration into a mixed species that cannot elongate to the desired SCP.[^38^, ^39^, ^40^, ^41^, ^42^, ^43^, ^44^] This leads to a decrease in the concentration of monomers prone to stack into SCP, and thus to a lower thermodynamic stability of the resulting SCP as reflected by higher critical concentration of aggregation (c*) and lower temperature of elongation (*Te*).^[45]^ We report herein that it is possible to engineer the coupled equilibria of the species involved in the coassembly process by a minimal change in the chemical nature of one of the interacting monomers. The principle is schematized in Scheme 1a for disk‐like monomers and implemented with BTA monomers and assemblies in apolar solvents (Scheme 1b). Despite being structurally‐similar, the chiral monomer (or “sergeant”) derived from amino‐ether (**BTA** **Eth**, red disk) yields significantly more stable SCP than the chiral monomer derived from amino‐ester (**BTA** **Est**, orange disk). Improved thermodynamic stability is demonstrated for achiral non‐functionalized and phosphine‐functionalized BTA monomers (the “soldiers”) affording better rheological and catalytic properties to the corresponding supramolecular copolymers.

## Results and Discussion

**Figure 1:**
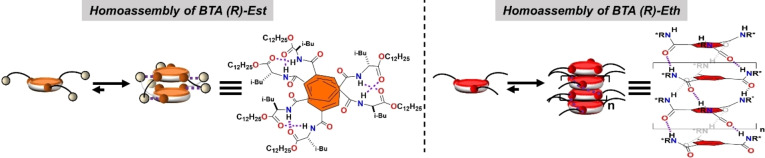
Structure of the homoassemblies. Schematic representation of the homoassemblies formed by **BTA** **(*R*)**–**Est** and by **BTA** **(*R*)**–**Est** as determined by FT‐IR, CD, and DOSY analyses performed in toluene and MCH (Figure S1–S4, Tables S1–S2).

**Selection and synthesis of the BTA monomers**. **BTA** **Est**, the BTA derived from the dodecyl ester of Leucine, was previously combined to **BTA** **P** coordinated to copper in order to yield enantioselective supramolecular helical catalysts (Scheme 1b).[^34^, ^46^, ^47^] **BTA** **Est** efficiently intercalates into the stacks formed by **BTA** **P** at relatively high concentrations,^[34]^ despite the fact that it forms dimers on its own.^[49]^ These dimers have been well‐characterized by our group (see a representation in Figure 1):[^48^, ^49^, ^50^, ^51^] the N−H moieties are hydrogen bonded to ester C=O, thus preventing the formation of amide‐bonded stacks for homoassemblies, even at high concentrations. In the case of coassemblies, these **BTA** **Est** dimers and potentially mixed species involving ester as hydrogen‐bond acceptors form in competition with the desired copolymers (stacks) embedding the amide‐bonded monomers.^[40]^ We postulated that designing a “sergeant” with a different type of hydrogen bond acceptor in its side chain will affect the stability of the competing species. We thus targeted a “sergeant” in which the ester carbonyl was replaced by a methylene. The (*R,R,R)* and (*S,S,S)* enantiomers of **BTA** **Eth** were synthesized in four steps (ca. 30 % overall yield) from commercially available Leucinol and isolated in their enantiopure forms as indicated by chiral HPLC analyses (Supporting Information).

**Characterization of the homoassemblies** (Figure 1). Concentration‐dependent FT‐IR, CD, and NMR analyses were performed to identify the structure of the homoassemblies formed by **BTA** **Est** and **BTA** **Eth** in toluene (Figures S1–S4). All these analyses confirm that **BTA** **Est** forms ester‐bonded dimers as the dominant species over a large range of concentrations. On the contrary, **BTA** **Eth** monomers convert into amide‐bonded helical stacks upon increasing the concentration as indicated by FT‐IR and CD signals characteristic of these species (Figures S1a and S2a, resp.). DOSY analyses show that the helical stacks formed by **BTA** **Eth** above 5.8 mM exhibit lower diffusion coefficients than **BTA** **Est** dimers and allow to determine a lower limit value of the degree of polymerization of 9 (30.5 mM). Likewise, Isothermal Titration Calorimetry (ITC) analyses were conducted to probe the stability of these homoassemblies upon dilution (Figure S5). Dimers of **BTA** **Est** are extremely stable (c*<0.04 mM), in agreement with our previous investigation.^[48]^ The critical concentration for the formation of stacks of **BTA** **Eth** is of ca. 5 mM. Finally, solutions of **BTA** **Eth** and **BTA** **Est** (mM range) are viscous and non‐viscous, respectively, a direct consequence of their polymeric and discrete nature (Figure S6). The sudden increase of viscosity for **BTA** **Eth** solutions upon raising the concentration is consistent with a cooperative mechanism of association which is a hallmark of BTA assemblies.^[52]^ Based on literature data, it was not totally obvious whether **BTA** **Eth** could form helical stacks on its own because of the hydrogen‐bond accepting ability of the ether functions.^[53]^ Our data unambiguously reveal that: (i) **BTA** **Eth** does not form dimers probably because of the unfavourable geometry of the potentially‐formed hydrogen bonds involving the ether oxygen, (ii) **BTA** **Eth** aggregates into stacks, as a direct consequence of point (i), i.e., because of the suppression of species that compete with the formation of these helical stacks.

**Figure 2:**
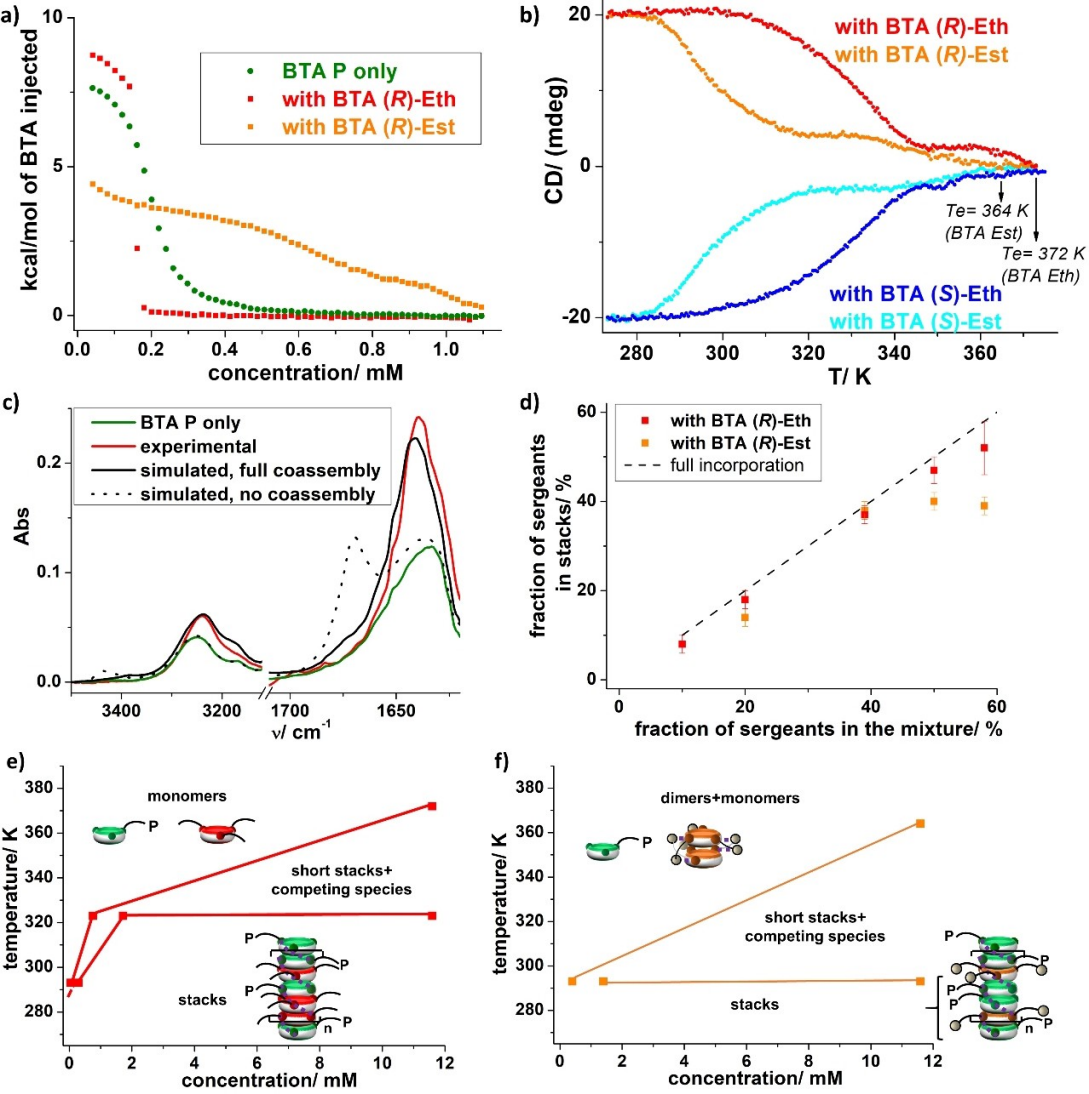
Probing the structure and stability of the coassemblies between **BTA** **P** and either **BTA** **Eth** or **BTA** **Est**. a) ITC enthalpograms obtained for toluene solutions containing **BTA** **P** alone, and mixtures of **BTA** **P** with either 50 % of **BTA** **(*R*)**–**Eth** or 50 % of **BTA** **(*R*)**–**Est** (total concentration in BTA=5.8 mM) injected into pure toluene, versus total BTA concentration in the cell at 293 K. ITC enthalpograms for homoassemblies and coassemblies have been compared in Figure S8. b) CD intensity (λ=295 nm) as a function of the temperature for the mixtures containing **BTA** **P** (5.8 mM) and one of the enantiomers of **BTA** **Eth** or **BTA** **Est** (5.8 mM). Data recorded upon heating (0.5 K . min^−1^). The elongation temperature (*Te*) was estimated at the onset of the rising of the CD signal. c) FT‐IR analyses of **BTA** **P** alone (5.8 mM) and of the mixture containing **BTA** **P** (5.8 mM) and 39 % of **BTA** **(*R*)**–**Eth** (3.7 mM). Zoom on the N−H and C=O regions. FT‐IR spectra for full and no coassembly have been simulated as indicated in the SI. d) Plot of the fraction of “sergeant” in the stacks (fs_s_) as a function of the fraction of “sergeant” initially introduced into the mixtures (fs_0_). e) Pseudo‐phase diagram (temperature versus concentration) for the coassemblies between **BTA** **P** and **BTA** **(*R*)**–**Eth** (1 : 1 mixture). Data were extracted from ITC (293 K and 323 K) and CD (11.6 mM). For CD, points are taken at the onset (i.e., *Te*) and 80 % of the plateau of the CD signal. f) Pseudo‐phase diagram for the coassemblies between **BTA** **P** and **BTA** **(*R*)**–**Est** (1 : 1 mixture). Data were extracted from ITC (293 K) and CD (11.6 mM). Competing species are small mixed species between **BTA** **P** and **BTA** **Est** or **BTA** **Eth** for which hydrogen bonding network does not exclusively involve amide C=O as acceptors but also ester or ether functions, respectively.

**Stability of the coassemblies**. The stability of the coassemblies formed by mixing an equimolar quantity of **BTA** **P** and **BTA** **(*R*)**–**Est** or **BTA** **(*R*)**–**Eth** (fs_0_=50 %) were firstly assessed by ITC, a powerful technique for this purpose.^[54]^ Solutions with a total concentration in BTA monomers of 5.8 mM were injected into a calorimetric cell filled with pure toluene at 293 K. After each injection, endothermal peaks are detected that correspond to the disruption of the hydrogen‐bonded species upon dilution. Once the critical concentration (c*) in the cell was reached, the stacks are stable enough and do not dissociate into monomers. Integration of the endothermal peaks obtained after each injection yields the enthalpograms shown in Figure 2a for which the enthalpy change (by mole of BTA injected), coming from the endothermic disruption of the assemblies, is plotted as a function of the total concentration in BTA. Solutions of **BTA** **P** alone and its mixture with **BTA** **(*R*)**–**Eth** exhibit similar ITC profiles: assemblies are fully disrupted at low concentration as can be deduced from the enthalpy change of the first injection of ca. 7–9 kcal . mol^−1^ consistent with the transition enthalpy between BTA stacks and monomers.^[48]^ Full assembly occurs at higher concentrations: critical concentrations (c*) can be extracted that correspond to twice the concentration at the mid‐point of the heat‐flow jump.^[55]^ This yields c* values of 0.35 mM and 0.40 mM for mixed stacks of **BTA** **P** and **BTA** **(*R*)**–**Eth** and homo stacks of **BTA** **P**, respectively. Small differences between the ITC traces of these assemblies will be commented below. The ITC profile exhibited by the solution containing the mixture between **BTA** **P** and **BTA** **(*R*)**–**Est** is drastically different. First, the enthalpy change for the first injections is roughly half of the expected dissociation enthalpy for BTAs, which is expected if disruption of the mixed stacks leads to **BTA** **P** monomers and **BTA** **(*R*)**–**Est** dimers, i.e., only half of all possible interactions are broken. Second, a very gradual decrease of the enthalpy change is observed and total coassembly is not fully reached at the highest measured concentration of 1.1 mM. This leads to an estimate value for c* of 1.4 mM. The gradual decrease likely indicates that competing species may form between **BTA** **P** and **BTA** **(*R*)**–**Est** at intermediate concentrations whilst dimers are the most likely predominant species at low concentration. Thus, these ITC analyses indicate that coassemblies with **BTA** **Eth** are far more stable thermodynamically than those of **BTA** **Est**, with a factor of 4 between their respective c* values at 293 K.

A similar trend is observed at 323 K: the c* is of 1.7 mM for coassemblies with **BTA** **Eth**, a concentration at which only competing species are present for **BTA** **Est** mixtures (Figure S7). Solutions with a smaller fraction of “sergeants” (fs_0_=20 %) have also been probed. The difference of stability is somewhat mitigated but remains significant: c*=0.35 mM and 0.60 mM for **BTA** **Eth** and **BTA** **Est** coassemblies, respectively (at 293 K).

The stability of coassemblies at a higher concentration has been probed by CD spectroscopy (Figure 2b). The evolution of the CD signal at λ=295 nm is monitored upon heating of the solutions containing the two types of coassemblies.^[56]^ The CD curves exhibit drastically different profiles from full coassembly at low temperature to disassembly at 373 K (no CD signal). Whilst the elongation temperature (*Te*) is only slightly higher for coassemblies with **BTA** **Eth** (372 K) than with **BTA** **Est** (364 K), the main difference comes from the fact that the curve for **BTA** **Est** coassemblies exhibits a non‐monotonic evolution with stable and constant CD signal observed only below 293 K. The evolution of CD signal for **BTA** **Eth** coassemblies indicates that stable coassemblies are maintained up to 310 K. Again, this confirms the higher stability of the BTA coassemblies integrating **BTA** **Eth** as “sergeant”. The non‐monotonic evolution of the CD signal may be attributed to competing species formed upon disassembly; they are clearly detected for **BTA** **Est** coassemblies and can also be surmised above 345 K, to a lesser extent, for the **BTA** **Eth** mixture.

**Rationalization of the enhanced stability of BTA Eth coassemblies**. Coassemblies were characterized by FT‐IR to elucidate the origin of the better stability observed when **BTA** **Eth** is used as “sergeant”. A constant amount of **BTA** **P** of 5.8 mM was selected to ensure sufficient coassembly for both “sergeants” into helical stacks. Whilst FT‐IR analyses appear relatively similar at moderate ratios of “sergeant” (fs_0_≤39 %), significant differences arise at higher ratios (Figure S9). For mixtures with **BTA** **Est**, signals corresponding to ester‐bonded dimers, and to potentially competing species with **BTA** **P**, are detected. Given the different signature between these species and stacks, it is possible to obtain the actual fraction of **BTA** **Est** incorporated into stacks (Figure S10). On the contrary, only stacks are observed for **BTA** **Eth** mixtures; all experimental curves compare well with those simulated for full coassembly of **BTA** **P** and **BTA** **Eth** in the same stacks (Figure 2c, and Figure S11). Accordingly, the plot of the fraction of “sergeant” in stacks (fs_s_) as a function of the fraction of “sergeant” initially introduced in the mixture (fs_0_) reveals that **BTA** **Eth** intercalates more efficiently into the stacks of **BTA** **P** than **BTA** **Est** (Figure 2d). **BTA** **Eth** actually has a far higher propensity to coassemble with **BTA** **P** than **BTA** **Est** and other BTAs previously investigated by our group.^[34]^

By combining this characterization of the composition of the helical coassemblies and their stability, the predominant species present under various conditions can be determined. The pseudo‐phase diagrams for coassemblies with **BTA** **Eth** and **BTA** **Est** shown in Figures 2e and 2 f, respectively, unambiguously indicate the higher stability of helical copolymers with **BTA** **Eth** since supramolecular copolymers (stacks) are the predominant species over a larger range of concentrations and temperatures. These diagrams also confirm that this better stability is mainly due to the fact that competing species (mixed species with **BTA** **P**) are minimized (or destabilized) with **BTA** **Eth** thus affording a more efficient intercalation with **BTA** **P** as depicted in Scheme 1a.

**Figure 3:**
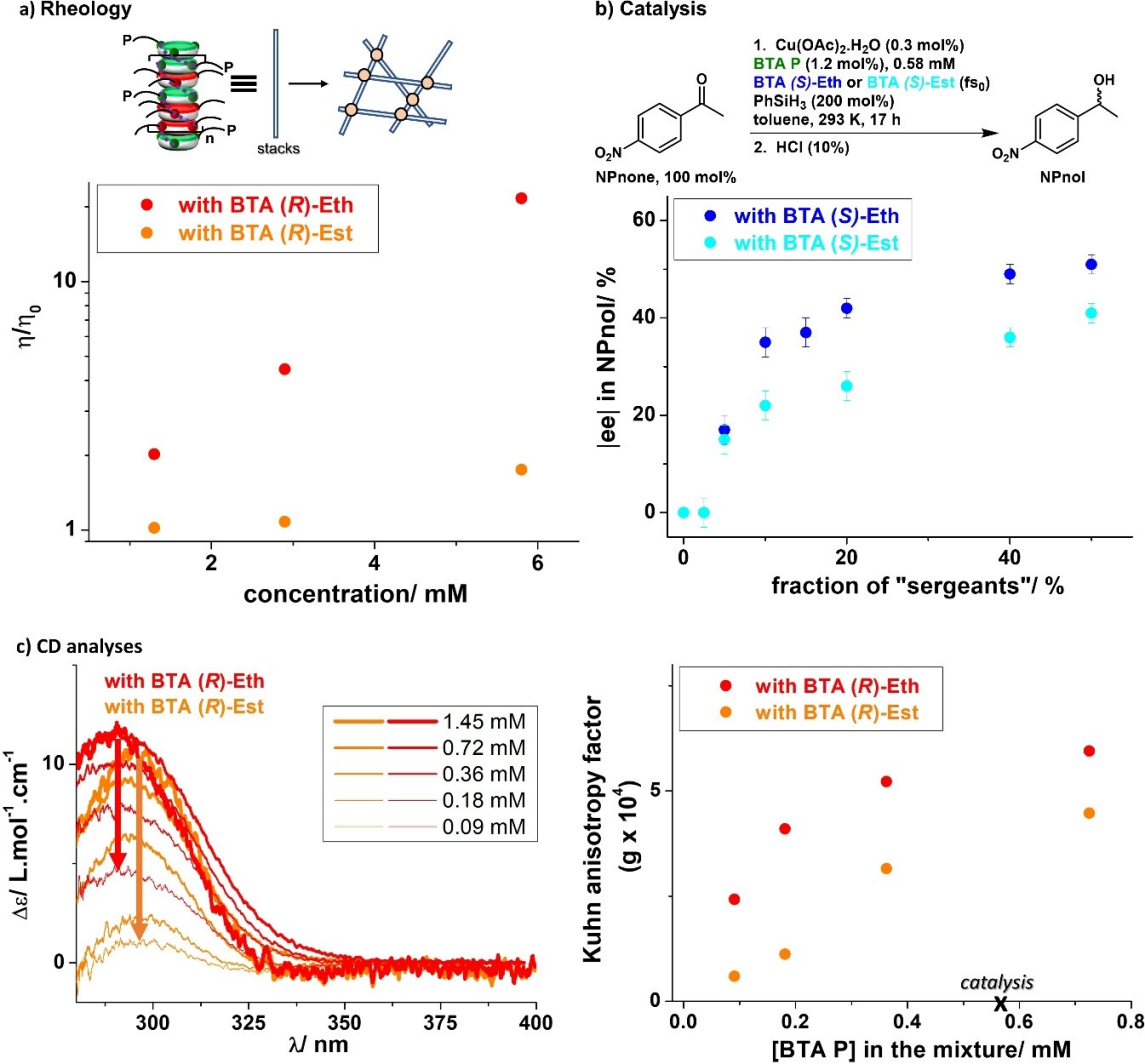
Macroscopic properties of the helical coassemblies. a) Relative viscosity for solutions containing **BTA** **P** and either 50 % of **BTA** **(*R*)**–**Eth** or 50 % of **BTA** **(*R*)**–**Est** in toluene at 293 K. The indicated concentration corresponds to the total concentration in BTA. Schematic representation of the entanglements (light orange spheres) between stacks constituted of **BTA** **P** and **BTA** **(*R*)**–**Et**. b) Enantiomeric excess (ee) in **NPnol** as a function of the fraction of “sergeants” (either **BTA** **Eth** or **BTA** **Est**) in the helical catalysts. Conversion >85 % (for fs_0_<10 %) and conversions >95 % (for fs_0_≥10 %). c) Left: CD spectra for solutions containing **BTA** **P** coordinated to copper (**BTA** **P**/[Cu]=4) and either 50 % of **BTA** **(*R*)**–**Eth** or 50 % of **BTA** **(*R*)**–**Est** at various concentrations in toluene at 293 K. The indicated concentration corresponds to that of **BTA** **P**. The molar extinction coefficient is calculated as Δϵ=θ/(32982×[**BTA** **P**]×l), with θ=ellipticity (in mdeg), [**BTA** **P**]=concentration in **BTA** **P** (in mol . L^−1^), and l=cell pathlength (in cm). Right: Kuhn anisotropy factor (g) as a function of the concentration of **BTA** **P** for mixtures containing **BTA** **P** coordinated to copper (**BTA** **P**/[Cu]=4) and either 50 % of **BTA** **(*R*)**–**Eth** or 50 % of **BTA** **(*R*)**–**Est**. The Kuhn anisotropy factor is determined as g=θ^295^/(32982×Abs^295^) where θ^295^ and Abs^295^ are the ellipticity and UV/Vis absorbance measured at λ=295 nm, respectively.

**Implications of the higher stability of the coassemblies at the macroscopic scale**. We compared the rheological and catalytic properties of helical coassemblies embedding **BTA** **P** as “soldier” and either **BTA** **Eth** or **BTA** **Est** as “sergeants”. Solutions with **BTA** **(*R*)**–**Eth** are far more viscous than those with **BTA** **(*R*)**–**Est** (Figure 3a). Stacks incorporating **BTA** **Eth** are thus longer, generating more entanglements. This is assumed to be a consequence of the better thermodynamic stability of their helical coassemblies (see also the discussion below).

Copper centres located at the periphery of the supramolecular helical copolymers can act as catalytic sites for an asymmetric reaction. Previous results from our group showed the ability of **BTA** **Est** helical coassemblies to induce a significant level of asymmetric induction in the copper‐catalysed hydrosilylation of 4‐nitroacetophenone (**NPnone**),^[34]^ the extent of which was related to the length of the helical stacks.^[57]^ Analytical data indicate that the presence of copper stabilizes coassemblies for both “sergeants” but those embedding **BTA** **Eth** are still significantly more stable (see Figures S12–S15 and the related discussion in the Supporting Information). In order to probe the robustness of the helical catalysts embedding **BTA** **Eth**, the total concentration in BTA monomers was divided 10 times relatively to our previously‐reported conditions. Catalytic mixtures with **BTA** **(*R*)**–**Eth** provide a higher enantiomeric excess in **NPnol** for all mixtures with fs_0_≥10 % (Figure 3b). In addition, only helical catalysts incorporating **BTA** **(*R*)**–**Eth** yield the optimal selectivity under these conditions (51±2 % ee). A selectivity of 78 % ee for a turnover number of ca. 300 was observed with a different BTA ligand at lower temperature, with still a significant advantage for the catalytic mixture with **BTA** **(*R*)**–**Eth** (Figure S16). At higher concentrations, i.e. under our classical conditions, both coassemblies exhibit similar catalytic properties (Figures S17–S18); this excludes a direct influence of the “sergeant” on the catalyst selectivity.

These catalytic results can actually be satisfactorily rationalized by analyzing diluted solutions of the coassemblies by CD. The CD signal at 295 nm decreases as the result of the disassembly of the helical copolymers, but this decrease is more gradual for **BTA** **(*R*)**–**Eth** containing mixtures, and a significant CD signal is still detected at the lowest investigated concentration of 0.09 mM (Figure 3c). At the concentration of the catalytic reaction (0.58 mM in **BTA** **P**), the difference is significant and consistent with the higher selectivity in **NPnol** observed with **BTA** **(*R*)**–**Eth** helical catalysts (Figure 3d). It is worth noting that the difference in CD intensity and catalytic selectivity at 0.58 mM is lower than expected relatively to the difference in stability reported in Figure 2. This may be attributed to either improved stability imparted by the presence of copper and/or the fact that small competing species involving **BTA** **P** monomers are able to promote the reaction with some enantioselectivity, yet not optimally. In overall, the increased stability of **BTA** **(*R*)**–**Eth** helical coassemblies enables to maintain optimal selectivity at a low catalytic loading (0.3 mol % of copper, i.e., one copper for 333 molecules of the substrate, **NPnone**).

**Figure 4:**
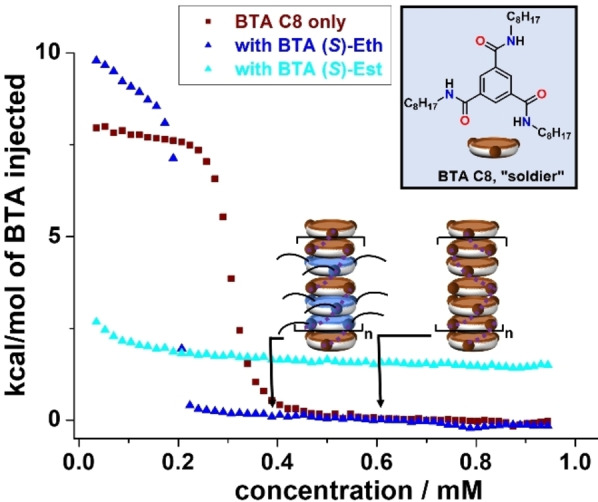
Supramolecular copolymerization with another “soldier”. ITC enthalpograms obtained for toluene solutions containing **BTA** **C8** alone, and mixtures of **BTA** **C8** with either 50 % of **BTA** **(*S*)**–**Eth** or 50 % of **BTA** **(*S*)**–**Est** (total concentration in BTA=5.0 mM) injected into pure toluene, versus total BTA concentration in the cell at 293 K. Critical concentrations for **BTA** **C8** only (c*=0.62 mM) and the coassemblies with **BTA** **(*S*)**–**Eth** (c*=0.40 mM) correspond to twice the concentrations at the mid‐point of the heat‐flow jump.^[56]^ Full coassembly is not reached for coassemblies with **BTA** **(*S*)**–**Est** at the highest concentration studied (0.95 mM). It leads to the estimate that the c* value is superior to 2 mM. In that case, the residual enthalpy (ca. 1.5 kcal . mol^−1^) at the highest measured concentration may correspond to the disassembly of short stacks or competing species between **BTA** **C8** and **BTA** **(*S*)**–**Est**.

**Further probing of the stabilization imparted by BTA Eth to helical coassemblies**. We were interested to probe whether the enhanced stability provided by **BTA** **Eth** relatively to **BTA** **Est** can be extended to another “soldier”. *N*,*N’*,*N*’’‐tris(octyl)benzene‐1,3,5‐tricarboxamide (**BTA** **C8**, Figure 4) was selected because of its *C*_3_ symmetry and its common use as “soldier” in BTA coassemblies.[^28^, ^32^, ^39^, ^40^, ^41^, ^44^, ^49^] ITC analyses in toluene indicate that homoassemblies of **BTA** **C8** are less stable than those of **BTA** **P** and thus the following stability order can be established for the single‐component stacks evaluated in this study: **BTA** **P** (c*=0.40 mM)>**BTA** **C8** (c*=0.62 mM)>**BTA** **(*S*)**–**Eth** (c*≈5 mM). More strikingly, coassemblies with **BTA** **(*S*)**–**Eth** are again significantly more stable than those with **BTA** **(*S*)**–**Est**, since c* values of 0.40 mM and superior to 2 mM, respectively, can be extracted from the ITC curves. It is expected that the main reason for the higher stability of the supramolecular coassemblies embedding **BTA** **Eth** and **BTA** **C8** is similar to the one demonstrated above for **BTA** **P**, i.e., that competing species between “sergeants” and “soldiers” are minimized. Residual enthalpy in the ITC curve at the highest concentration measured is indeed consistent with rather stable short stacks or competing species formed between **BTA** **Est** and **BTA** **C8** (Figure 4). Mixed trimer species, in which both ester and amide carbonyls participate in the hydrogen bonding network, have been postulated by Meijer and co‐workers as a possible structure for which a fraction of the “soldier” monomers are scavenged, thus limiting their growth into amide‐bonded stacks.^[40]^ With **BTA** **Eth**, most if not all of the BTA monomers are available for coassembly by stacking hence yielding SCP with higher stability.

Other factors leading to an extra stabilization of the coassemblies embedding **BTA** **Eth** cannot be discarded. A closer look at the ITC curves reveal that coassemblies with **BTA** **Eth** are actually more stable than the homoassemblies of the corresponding “soldiers” as shown by their lower c* values (Figures 2a and 4). A higher transition enthalpy is also observed for the coassemblies (9–10 kcal . mol^−1^) relatively to the homoassemblies (7–8 kcal . mol^−1^). One possible reason is that the helical coassemblies are homochiral (i.e., they are single handed) whereas homoassemblies of the “soldiers” are racemic (i.e., constituted of left‐handed and right‐handed fragments as represented for **BTA** **C8** in Figure 4). The removal of chiral defects might indeed contribute to the stabilization of the coassemblies.^[37]^ In addition, the free energy of heterointeraction (between **BTA** **Eth** and the “soldier”) may be higher than the free energy of homointeractions (between “soldiers” and between “sergeants”).^[24]^ This last point would constitute an additional factor contributing to the favourable intercalation of **BTA** **Eth** into the stacks of the “soldiers”, as well as to the formation of long SCP; these two properties are consistent with our experimental observations (Figures 2d and 3a).

## Conclusions

Herein, we demonstrate that a simple modification in the chemical structure of the chiral monomer, used as “sergeant”, has drastic influence on its coassemblies formed with an achiral monomer, the “soldier”. The replacement of an ester by an ether function leads to a significant stabilization of the corresponding supramolecular copolymers, mainly as a consequence of the minimization (or destabilization) of competing species, i.e. small species that scavenge a fraction of the monomers into non‐desired supramolecular structures. This principle is operative for several “soldiers”, including BTA ligands coordinated to copper. A good dynamicity of the supramolecular copolymers is maintained which in turn demonstrate better rheological and catalytic properties. It complements well literature precedents in which reversible cross‐links^[58]^ or covalent trapping^[59]^ of the side chains have been exploited to enhance supramolecular (co)polymer stability. The present concept has been demonstrated for benzene‐1,3,5‐tricarboxamide (BTA) monomers, one of the most widely used synthons in supramolecular chemistry, but it can potentially be extended to other families of monomers, notably the large family of monomers with a disc‐like geometry^[60]^ as well as for assemblies exhibiting polymorphism^[9]^ by proper engineering of the multiple types of non‐covalent interactions engaged between the monomers.

## Supporting Information

The authors have cited additional references within the Supporting Information.[^61^, ^62^, ^63^, ^64^, ^65^, ^66^, ^67^]

## Conflict of Interests

The authors declare no conflict of interest.

1

## Supporting information

As a service to our authors and readers, this journal provides supporting information supplied by the authors. Such materials are peer reviewed and may be re‐organized for online delivery, but are not copy‐edited or typeset. Technical support issues arising from supporting information (other than missing files) should be addressed to the authors.

Supporting Information

## Data Availability

The data that support the findings of this study are available in the supplementary material of this article.
