# Supplementary material for: Enhanced Stability and Properties of Benzene‐1,3,5‐Tricarboxamide Supramolecular Copolymers through Engineered Coupled Equilibria
Source: Angew Chem Int Ed Engl. 2024 Dec 8;64(12):e202421991. doi: 10.1002/anie.202421991 (PMC11914932; doi:10.1002/anie.202421991)
Supplement: Supplementary file 1 — Supporting Information [file ANIE-64-e202421991-s001.pdf]

Angewandte Chemie
Eine Zeitschrift der Gesellschaft Deutscher Chemiker

Supporting Information

Enhanced Stability and Properties of Benzene-1,3,5-Tricarboxamide Supramolecular Copolymers through Engineered Coupled Equilibria

*H. Kong, A. Valverde-González, R. Maruchenko, L. Bouteiller, M. Raynal**

Supporting information

Enhanced Stability and Properties of Benzene-1,3,5-tricarboxamide Supramolecular Copolymers through Engineered Coupled Equilibria

Huanjun Kong, Antonio Valverde-González, Régina Maruchenko, Laurent Bouteiller, and Matthieu Raynal*

† Sorbonne Université, CNRS, Institut Parisien de Chimie Moléculaire, 4 Place Jussieu, 75005 Paris (France)
e-mail: matthieu.raynal@sorbonne-universite.fr.

Supplementary Chart S1, Figures S1-S18 and Tables S1-S2 ______ 2

General methods ______ 21

Catalytic experiments, Tables S3-S6 ______ 25

Solutions for ITC, NMR, FT-IR, CD and rheology analyses ______ 29

Synthesis of BTA Eth, Figures S19-S23 ______ 30

Optical purity of **BTA (*S*)-Eth** and **BTA (*R*)-Eth**, Figure S24 ______ 35

Selected chiral GC analyses, Figures S25-S37 ______ 36

References ______ 43

Supplementary Chart S1, Figures S1-S18 and Tables S1-S2

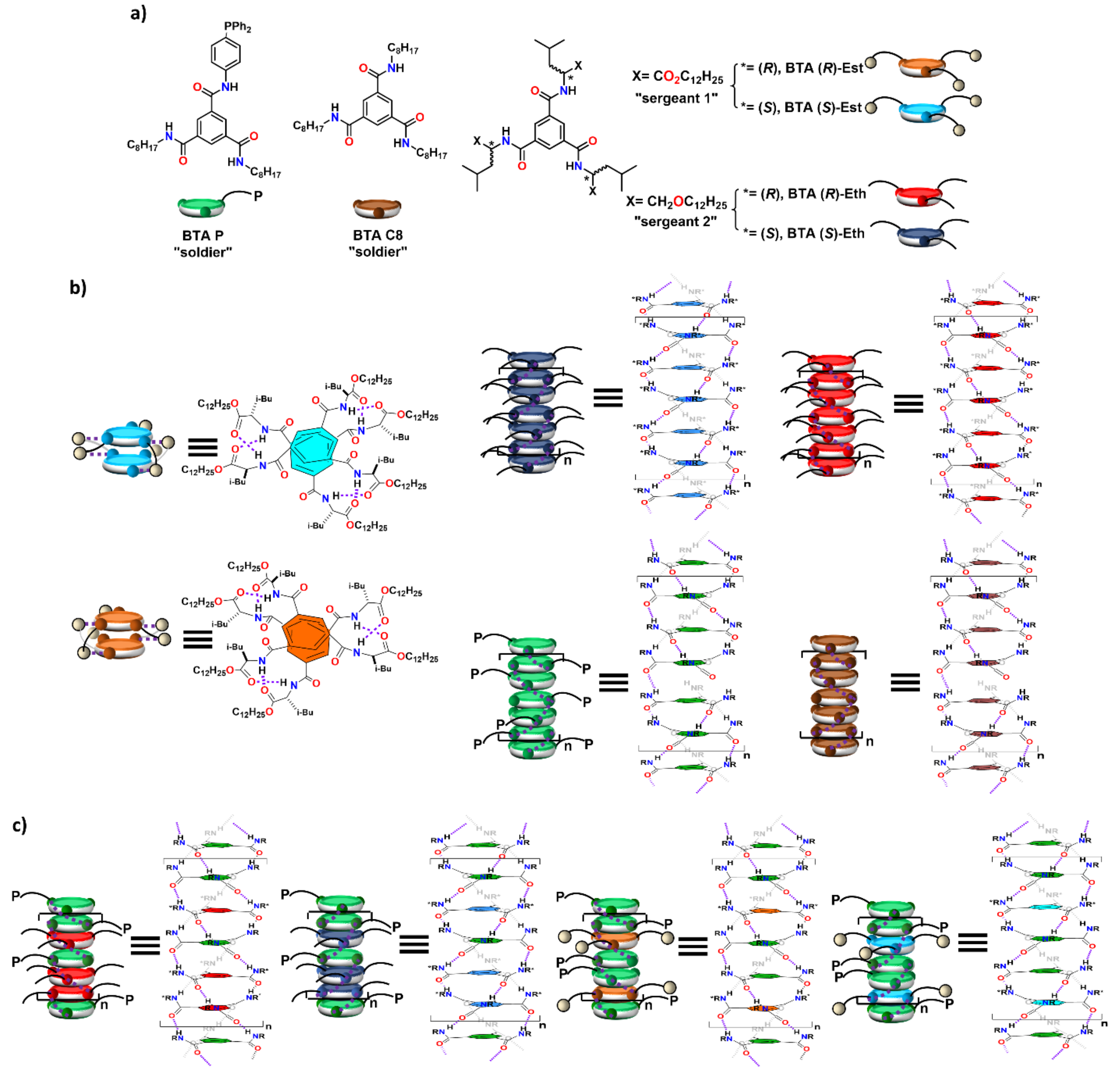

Chart S1. a) Chemical structures of BTA monomers used in this study ("soldiers" and "sergeants" are achiral and chiral monomers, respectively). b) Structure of the homoassemblies formed by **BTA (*R*)-Est** (dimer), **BTA (*S*)-Est** (dimer), **BTA (*R*)-Eth** (stacks), **BTA (*S*)-Eth** (stacks), **BTA P** (stacks) and **BTA C8** (stacks). c) Structure of the coassemblies/copolymers formed by mixing **BTA P** with either **BTA (*R*)-Est**, **BTA (*S*)-Est**, **BTA (*R*)-Eth**, or **BTA (*S*)-Eth** (competing species and dimers are not represented). The handedness of the helices is represented as dotted lines.

Convention: Throughout this paper, the fraction of enantiopure BTA monomers (or "sergeants") initially introduced in the BTA mixture (fs_0), and the fraction of enantiopure BTA monomers (or "sergeants") present in BTA stacks (fs_S) are defined as follows: fs_0= ["sergeant"]/(["sergeant"]+["soldier"]) and fs_s= ["sergeant in stacks"]/(["sergeant in stacks"]+["soldier"]), respectively. "Soldier"= **BTA P** or **BTA C8**.

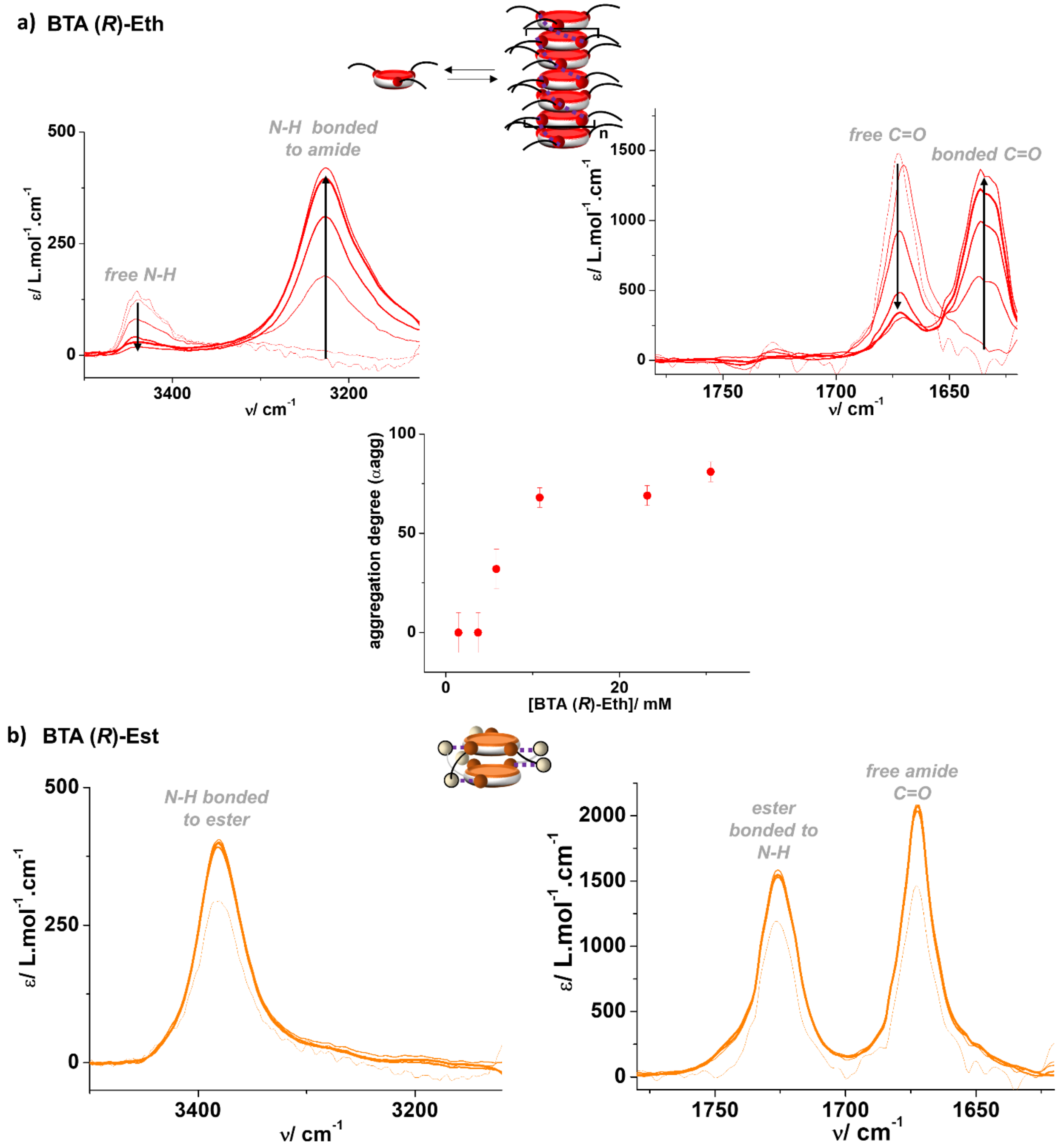

Figure S1. FT-IR analyses of **BTA (*R*)-Eth** (a) and **BTA (*R*)-Est** (b) at various concentrations in toluene at 293 K (1.45 mM, 3.7 mM, 5.8 mM, 10.8 mM, 23.2 mM for both and 30.5 mM in addition for **BTA (*R*)-Eth** only). Zoom on the N─H and C=O regions. Arrows show the evolution of the FT-IR bands upon increasing the concentration. Aggregation degree for **BTA (*R*)-Eth** is calculated by considering that only monomers are present in the most diluted solution (1.45 mM).

Interpretation: Stretching frequencies at the maximum of the FT-IR bands are consistent with the attribution of monomers/stacks [free N─H: 3438 cm^{-1}, N─H bonded to amide: 3226 cm^{-1}, free amide C=O: 1672 cm^{-1}, bonded amide C=O: 1636 cm^{-1}] and dimers [N─H bonded to ester: 3381 cm^{-1}, bonded ester C=O: 1726 cm^{-1}, free amide C=O: 1673 cm^{-1}].[1-3]

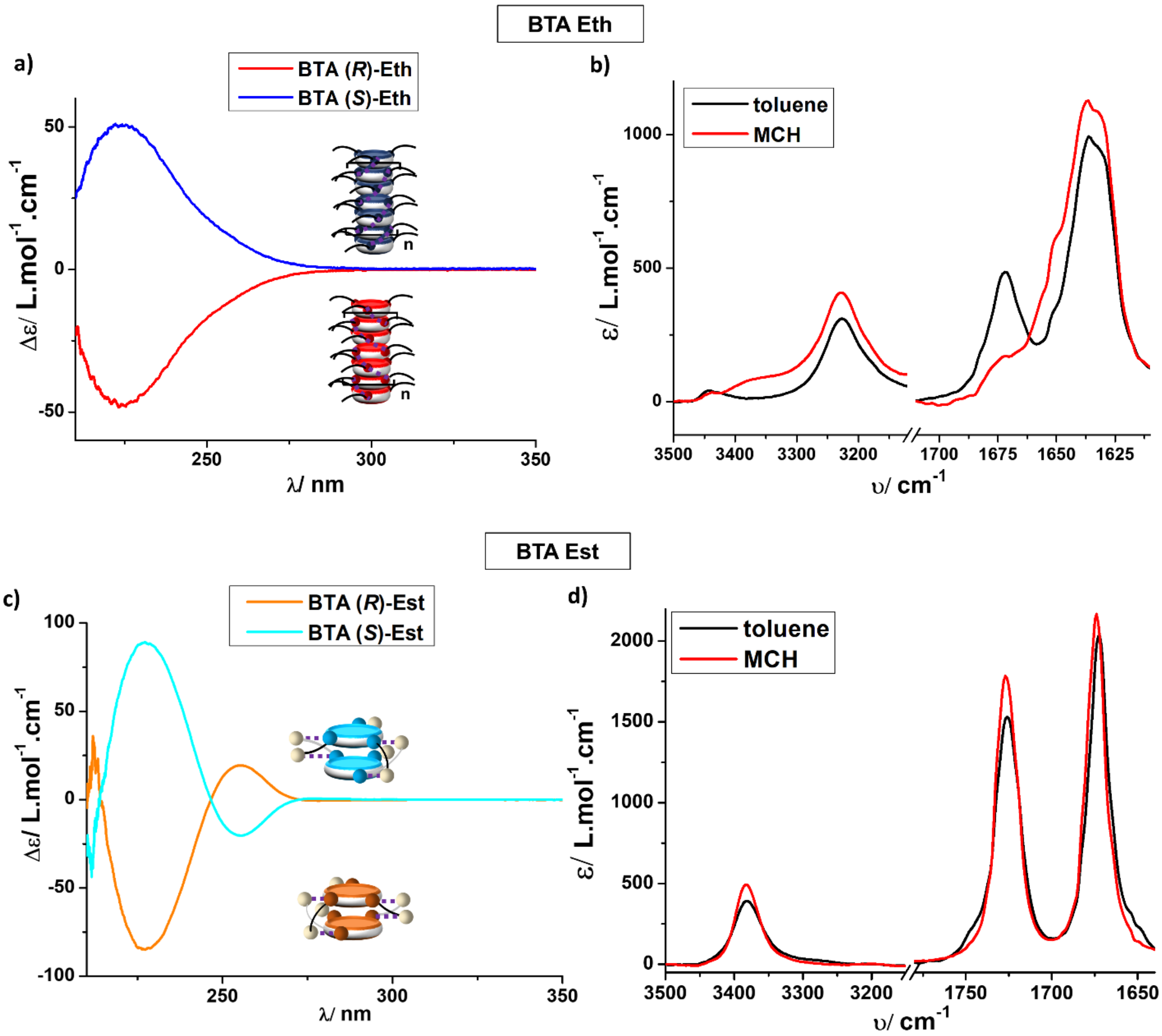

Figure S2. CD analyses of **BTA (*R*)-Eth** and **BTA (*S*)-Eth** (a) and of **BTA (*R*)-Est** and **BTA (*S*)-Est** (c) in methylcyclohexane at 293 K (10 mM). FT-IR analyses (zoom on the N─H and C=O regions) of **BTA (*R*)-Eth** (b) and **BTA (*R*)-Est** (d) in toluene (10.8 mM) and MCH (10 mM).

Interpretation: CD analyses have been conducted in methylcyclohexane because the lower absorption of this solvent allows to get the CD fingerprint of the homopolymers. CD signals of BTA Eth and BTA Est have significantly different shape and intensities, in agreement with the presence of stacks and dimers, respectively.[1-3] CD spectra of both enantiomers are mirror images as expected for supramolecular structures with opposite handednesses. The sign and intensity of the CD band recorded for **BTA (*R*)-Eth** and **BTA (*S*)-Eth** are consistent with left-handed and right-handed homochiral stacks, respectively.[1-3] FT-IR analyses confirm that similar species are formed in both toluene and methylcyclohexane; **BTA Eth** being more aggregated in methylcyclohexane.

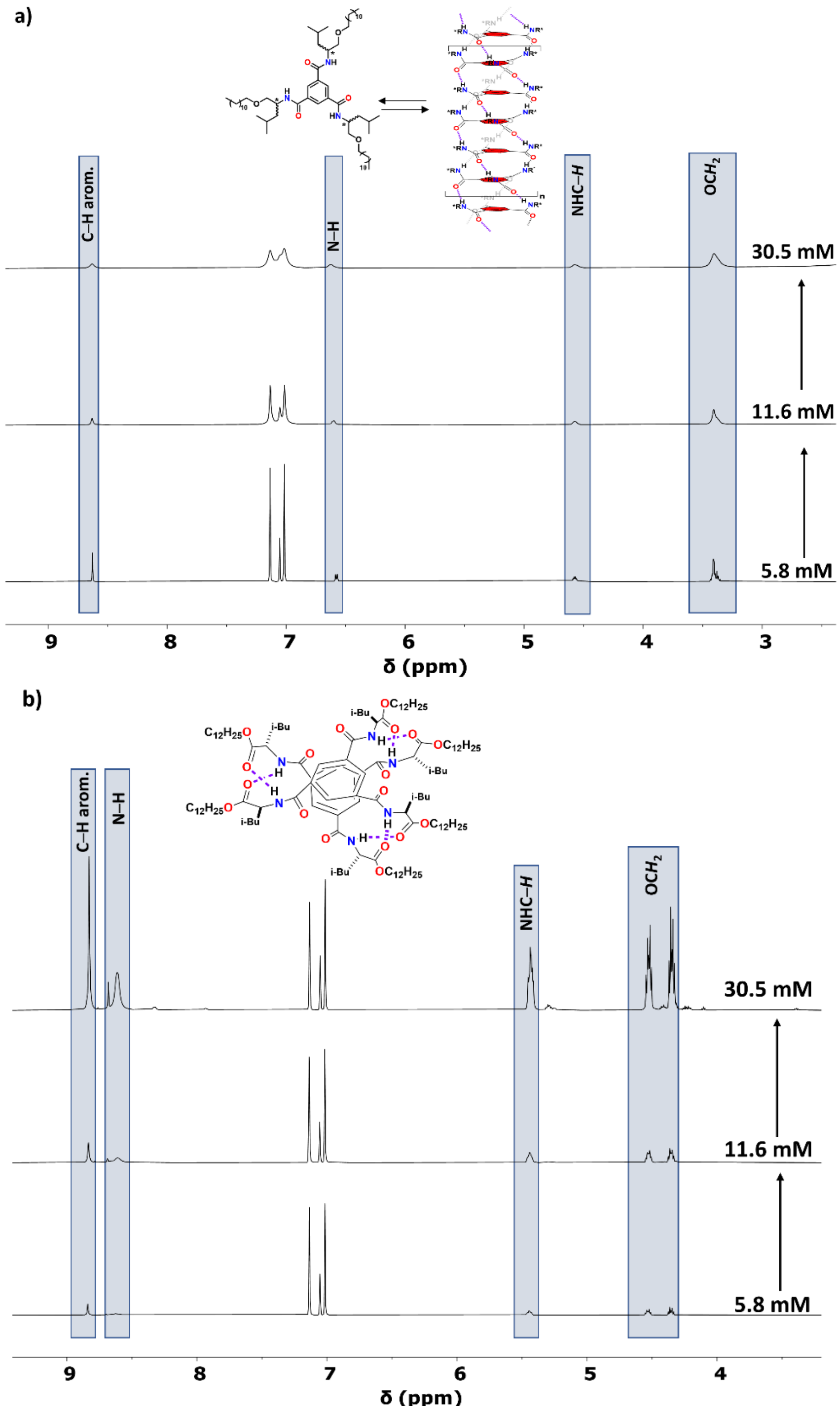

Figure S3. ^{1}H NMR analyses of **BTA (*R*)-Eth** and (a) and **BTA (*R*)-Est** (b) at various concentrations in C_7D_8 at 293 K (5.8 mM, 11.6 mM, 30.5 mM). Zoom on specific hydrogens. Residual toluene signals at 6.97, 7.01 and 7.09 ppm.

Interpretation: The well-resolved nature of the NMR signals and the fact that OCH_2 hydrogens are diastereotopic are consistent with the dimeric structure established for **BTA (*R*)-Est**.[1-3] For **BTA (*R*)-Eth**, all NMR signals, including those of the residual solvent, become broader upon increasing the concentration. This is attributed to the higher viscosity of these solutions (see Figure S6).

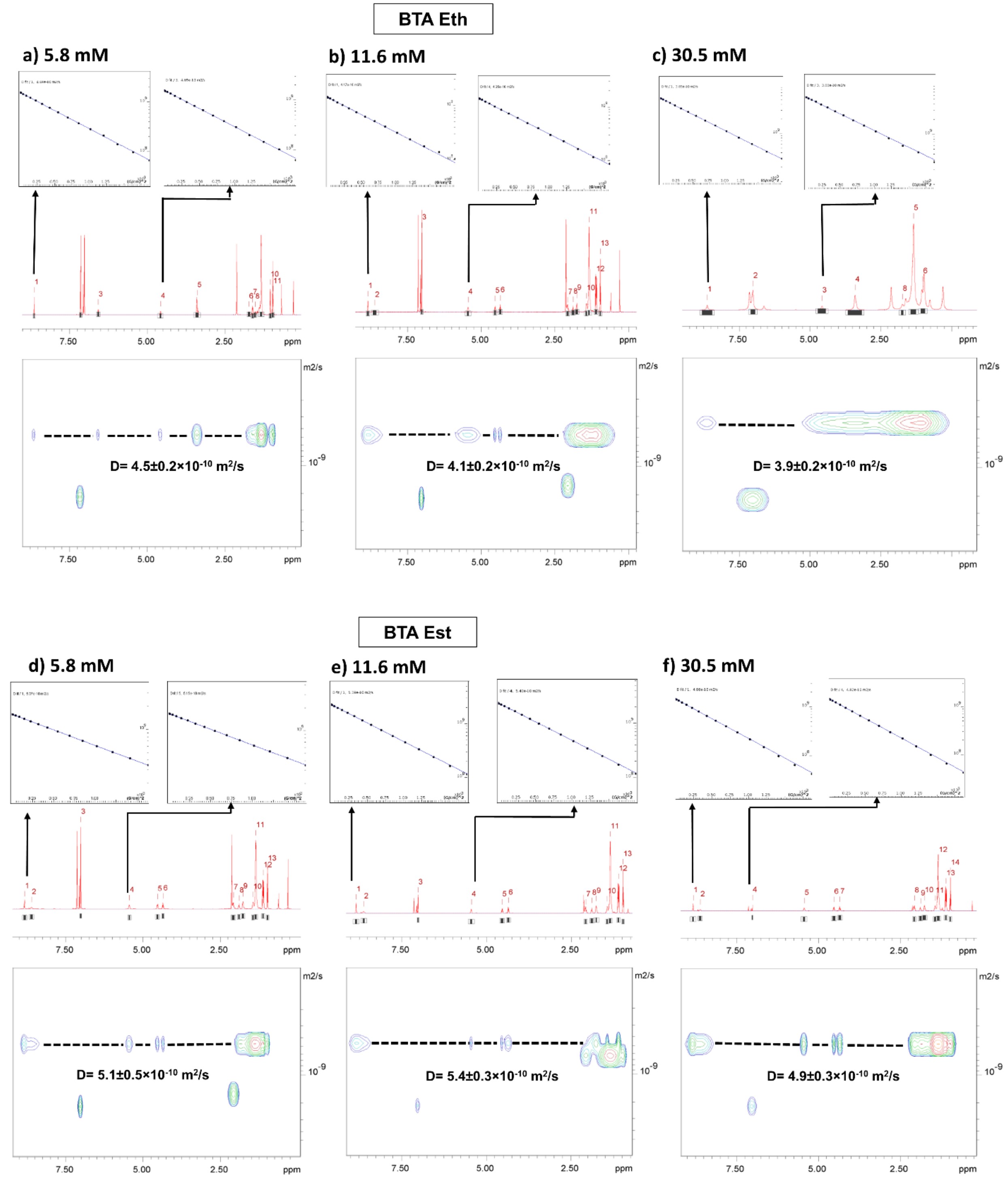

Figure S4. DOSY analyses of **BTA (*R*)-Eth** and (a-c) and **BTA (*R*)-Est** (d-f) at various concentrations in C_7D_8 at 293 K (5.8 mM, 11.6 mM, 30.5 mM). Monoexponential fitting is shown only for C─H arom. and NHC─*H* hydrogens. Indicated diffusion coefficients (with the corresponding error) correspond to average diffusion coefficients for all hydrogens. See the extracted geometrical parameters in Tables S1-S2.

**BTA (*R*)-Eth**	5.8 mM	11.6 mM	30.5 mM
D×10^{10} (m^2/s)	4.5±0.2	4.1±0.2	3.9±0.2
L (nm)	2.6	2.8	3.2
DP	6	8	9

Table S1. Diffusion coefficient (*D*), length (*L*), and polymerization degree (*DP*) extracted from DOSY experiments conducted with **BTA (*R*)-Eth** at various concentrations (Figure S4 a-c). Assuming the formation of rigid rods (consistently with FTIR and CD analyses), we deduce the length and *DP* values of the stacks from the diffusion coefficients *D* using the Tirado-Garcia de la Torre relation[4] (see reference [5]). We consider that these *DP* values are actually lower limit values for the stacks present in solution since it is highly probable that the larger species are not detected in the ^{1}H NMR spectra shown in Figures S3a and S4ac. It can be surmised that signals detected by NMR correspond to average NMR signals of the smaller species in rapid equilibrium on the NMR timescale (^{1}H NMR frequency).

**BTA (*R*)-Est**	5.8 mM	11.6 mM	30.5 mM
D×10^{10} (m^2/s)	5.1±0.5	5.4±0.3	4.9±0.3
r_h (Å)	8.6±0.7	8.3±0.3	9.0±0.5
V/Vm	2.2±0.5	2.0±0.2	2.5±0.5

Table S2. Diffusion coefficient (*D*), hydrodynamic radius (r_h), and ratio between the volume of the spherical assemblies (*V*) and the volume of the monomer (*Vm*) extracted from DOSY experiments conducted with **BTA (*R*)-Est** at various concentrations (Figure S4 d-e). The hydrodynamic radius was deduced from the diffusion coefficients *D* using the Stokes-Einstein equation, valid for spherical objects such as those formed by **BTA (*R*)-Est**. The hydrodynamic radius of the monomer was obtained from the DOSY analysis (data not shown) of ^{Me}BTA Val, a BTA molecule with methylated amide function unable to assemble.[2] The value obtained for the monomer in C_7D_8 (r_h= 6.6 Å) is close to the value previously obtained in C_6D_{12} (r_h= 7.1 Å).[2] The *V*/*Vm* values are consistent with the presence of dimers whatever the concentration.

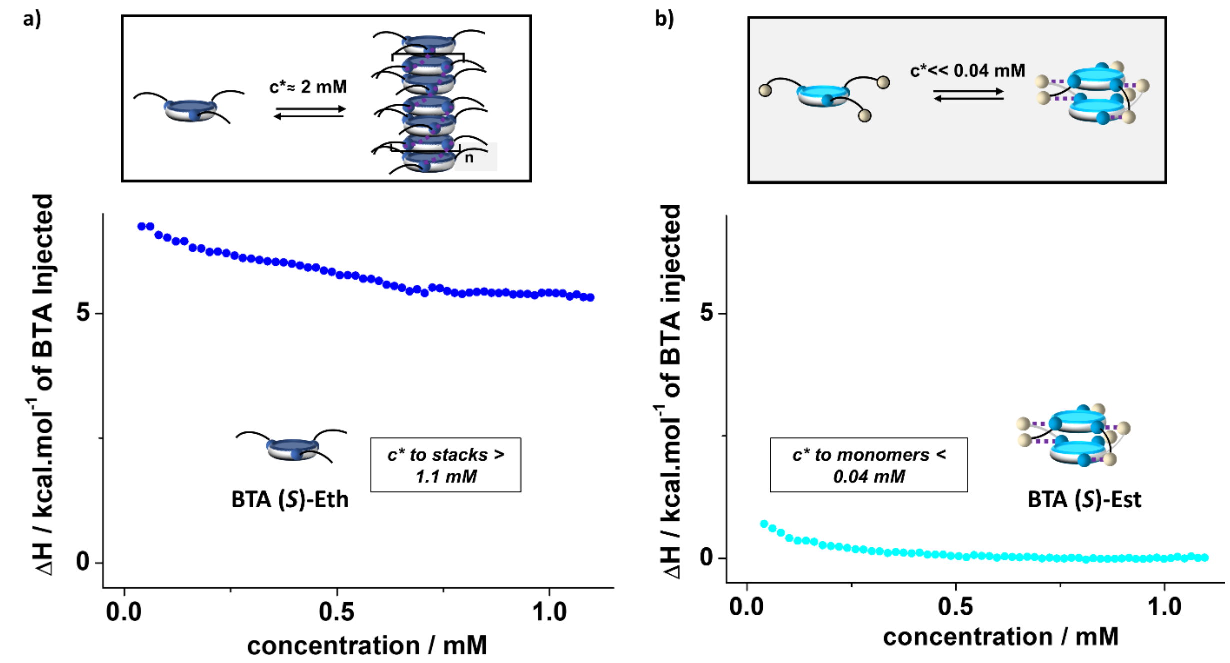

Figure S5. ITC enthalpograms obtained for solutions of **BTA (*S*)-Eth** (a) and **BTA (*S*)-Est** (b) in toluene (5.8 mM) injected into pure toluene, versus total BTA concentration in the cell at 293 K.

Interpretation: a) Stacks of **BTA (*S*)-Eth** dissociate up to the measured concentration of 1.1 mM (ΔH = 6 kcal/mol). This is consistent with FT-IR data shown in Figure S1a, where a critical concentration of *ca.* 5 mM can be deduced for the stacks of **BTA Eth**. b) The heat of dissociation is negligible, which indicates that dimers of **BTA (*S*)-Est** are still mostly formed even at the lowest concentration of 0.04 mM. This means that the critical concentration of the dimers of **BTA Est** is below 0.04 mM.

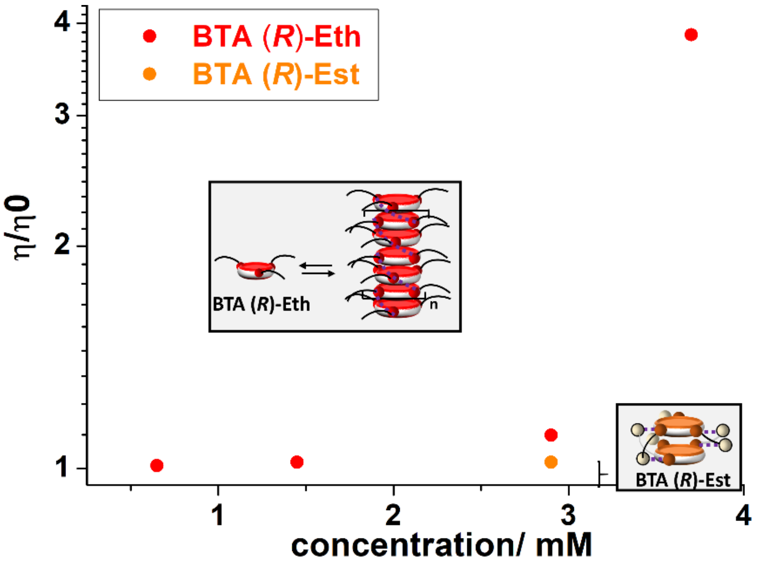

Figure S6. Relative viscosity for solutions of **BTA (*R*)-Eth** and **BTA (*R*)-Est** at various concentrations in toluene at 293 K.

Interpretation: **BTA (*R*)-Eth** forms stacks that are sufficiently long at 3.7 mM to entangle and slow down the flow of the solution whilst **BTA (*R*)-Est** forms dimers.

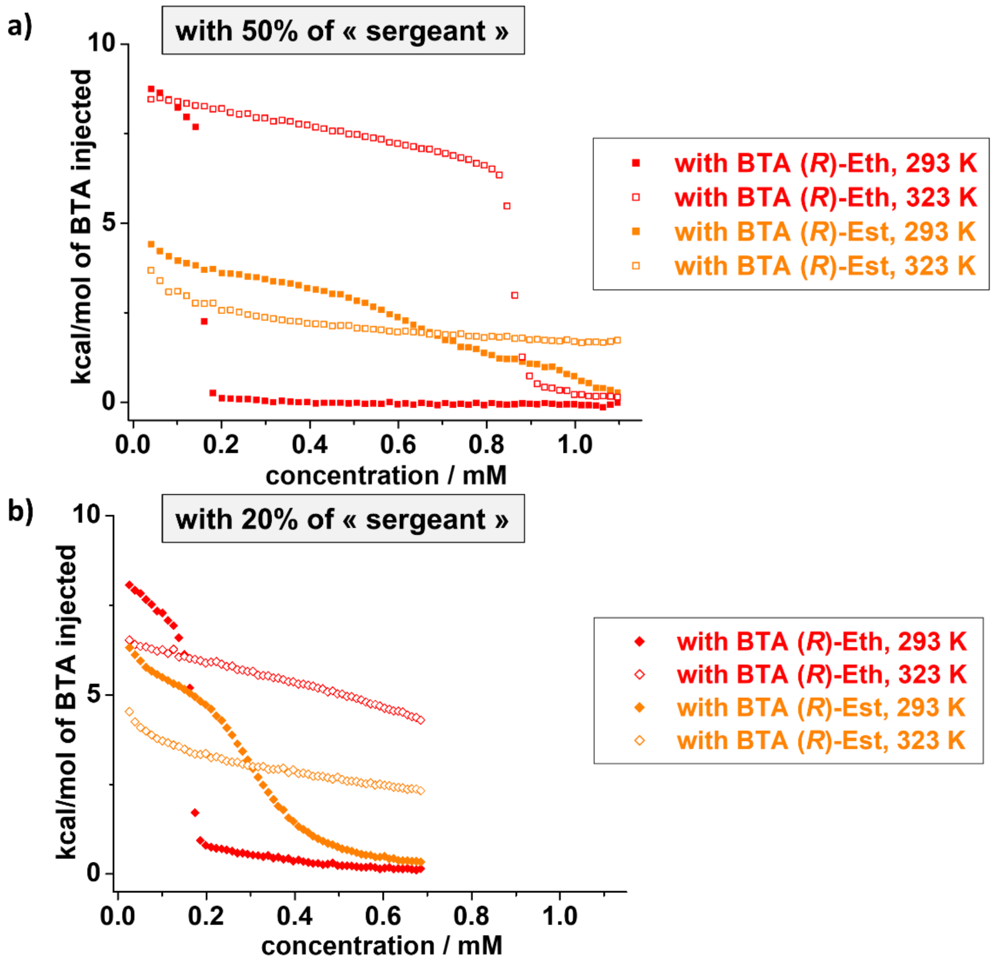

Figure S7. a) ITC enthalpograms obtained for solutions containing **BTA P** and either 50% of **BTA (*R*)-Eth** or 50% of **BTA (*R*)-Est** in toluene (total concentration in BTA= 5.8 mM) injected into pure toluene, versus total BTA concentration in the cell at 293 K or 323 K. b) ITC enthalpograms obtained for solutions of mixtures containing **BTA P** and either 20% of **BTA (*R*)-Eth** or 20% of **BTA (*R*)-Est** in toluene (total concentration in BTA= 3.6 mM) injected into pure toluene, versus total BTA concentration in the cell at 293 K or 323 K. Critical concentrations cannot be precisely determined for all mixtures with **BTA (*R*)-Est** at 323 K and for the mixture with 20% of **BTA (*R*)-Eth** at 323 K due to the fact that full coassembly is not reached at the highest concentration in the cell.

For the other mixtures, the following critical concentrations are determined (critical concentrations correspond to twice the concentration at the mid-point of the heat-flow jump according to reference [2]):

BTA P + **BTA (*R*)-Eth** (fs_0= 50%), 293 K, *c**= 0.35 mM.

BTA P + **BTA (*R*)-Est** (fs_0= 50%), 293 K, *c**= 1.4 mM.

BTA P + **BTA (*R*)-Eth** (fs_0= 50%), 323 K, *c**= 1.7 mM.

BTA P + **BTA (*R*)-Eth** (fs_0= 20%), 293 K, *c**= 0.35 mM.

BTA P + **BTA (*R*)-Est** (fs_0= 20%), 293 K, *c**= 0.60 mM.

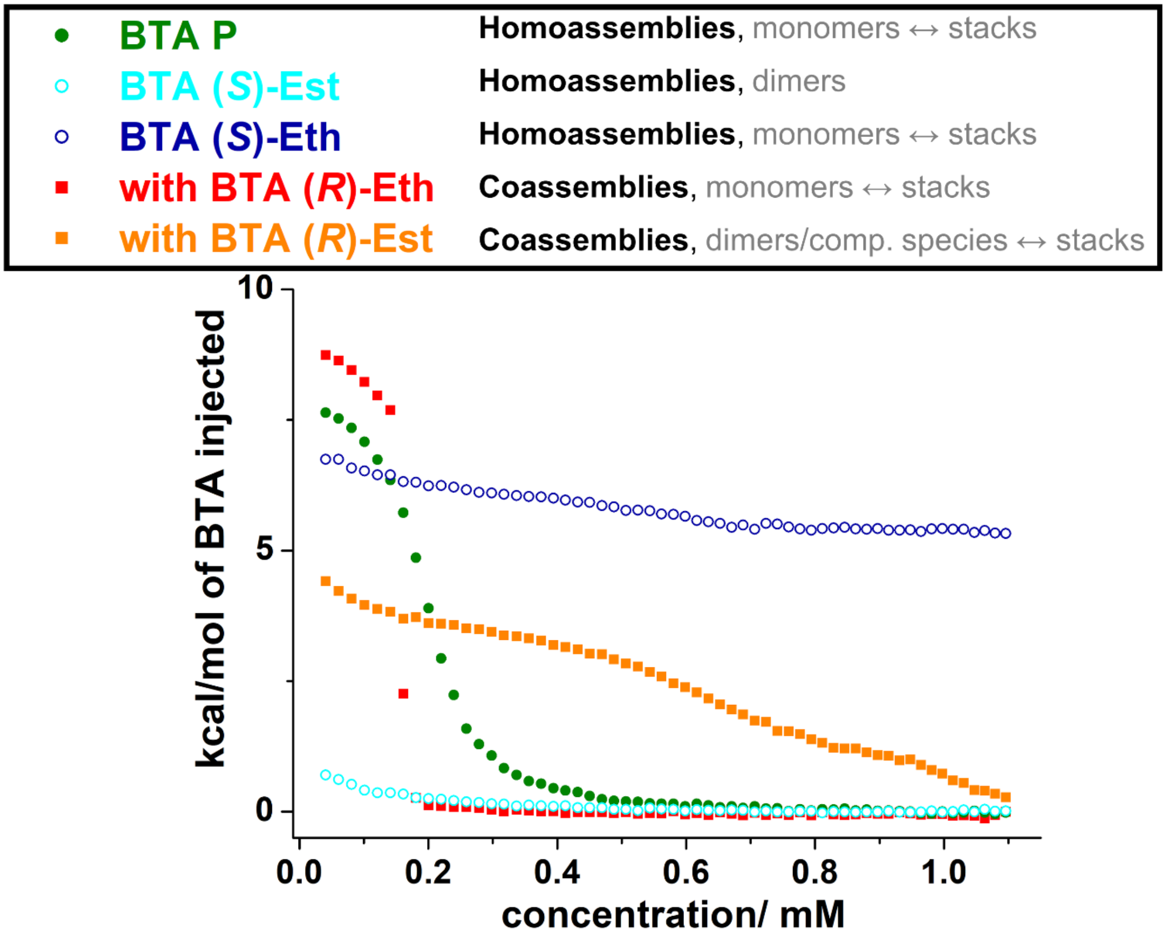

Figure S8. Compared ITC enthalpograms for homoassemblies of **BTA P**, **BTA (*S*)-Est** and **BTA (*S*)-Eth** and 1:1 coassemblies between **BTA P** and either **BTA (*R*)-Eth** or **BTA (*R*)-Est** in toluene (total concentration in BTA= 5.8 mM).

The different species involved in the (dis)assembly process are indicated in the legend:

BTA P *c**= 0.40 mM (monomers ↔ stacks)

BTA (*S*)-Est c*< 0.040 mM (monomers ↔ dimers)

BTA (*S*)-Eth *c**≈ 5 mM (monomers ↔ stacks)

BTA P + **BTA (*R*)-Eth** *c**= 0.35 mM (monomers ↔ stacks)

BTA P + **BTA (*R*)-Est** *c**= 1.4 mM (dimers/competing species ↔ stacks)

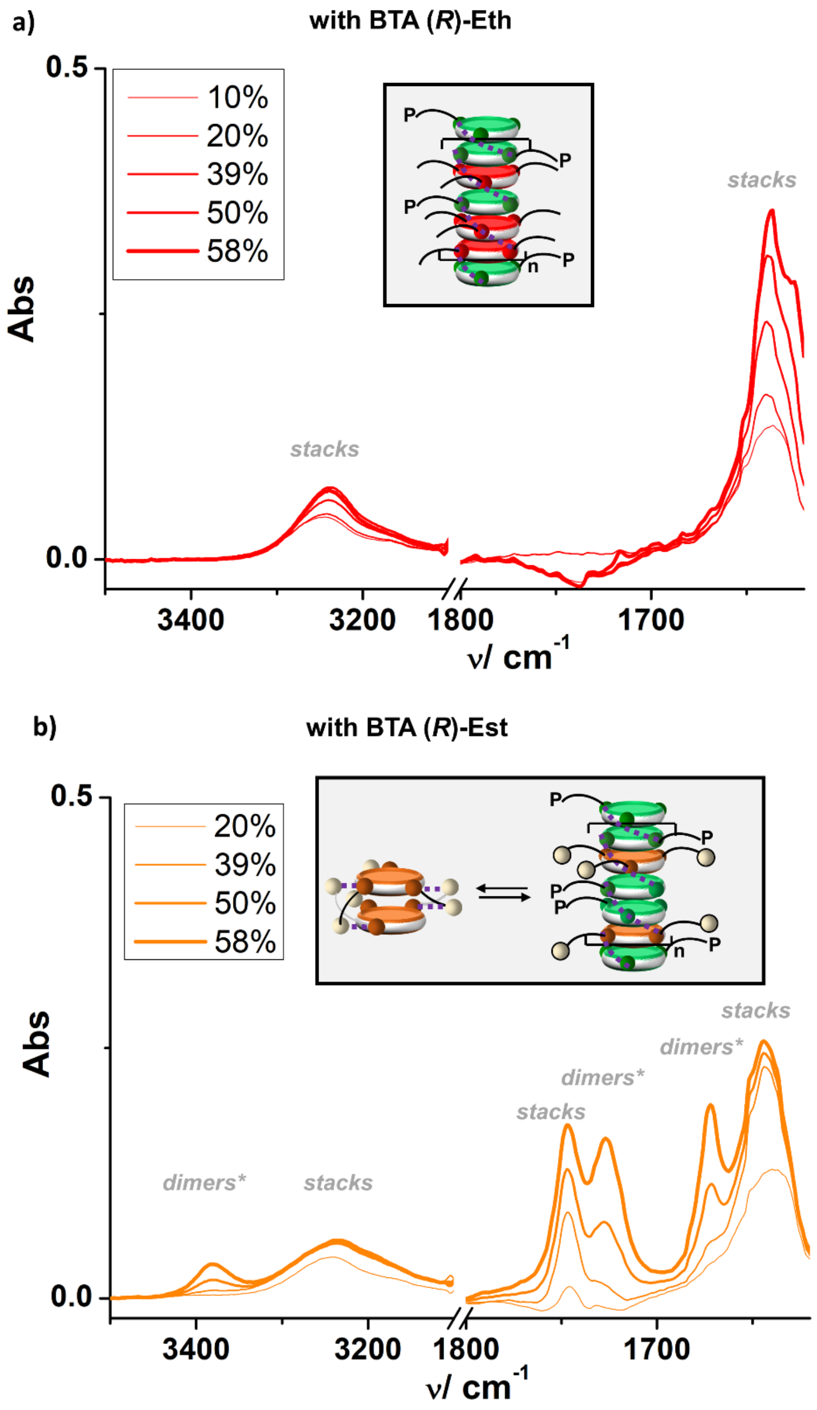

Figure S9. FT-IR analyses of the "sergeants-and-soldiers"-type mixtures containing **BTA P** (5.8 mM) and either **BTA (*R*)-Eth** (0.65 mM─8.0 mM, 10%≤ fs_0≤ 58%) or **BTA (*R*)-Est** (1.45 mM─8.0 mM, 20%≤ fs_0≤ 58%) in toluene at 293 K. Zoom on the N─H and C=O regions. Dimers*= these bands can also include competing species between **BTA P** and **BTA (*R*)-Est** (not stacks) for which N─H can be bonded to ester and amide C=O.[2,6]

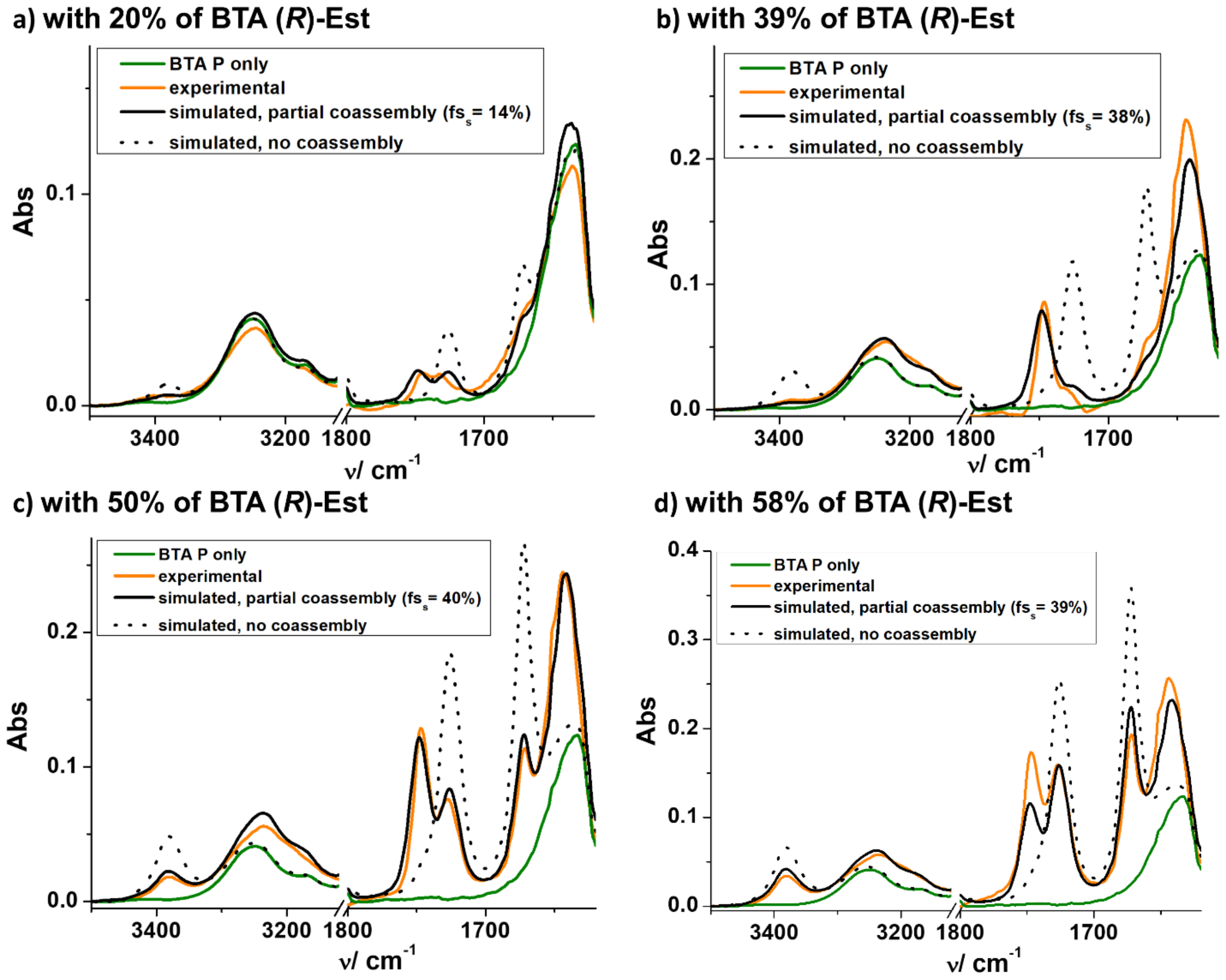

Figure S10. FT-IR analyses of the “sergeants-and-soldiers”-type mixtures containing **BTA P** (5.8 mM) and **BTA (*R*)-Est** (1.45 mM─8.0 mM, 20%≤ fs$_0$≤ 58%) in toluene at 293 K. Zoom on the N─H and C=O regions. The FT-IR spectrum for **BTA P** only (5.8 mM) is also shown. FT-IR spectra for full and no coassembly have been simulated as indicated in the General Methods. For the attribution of the bands, see Figure S9. Experimental spectrum for the mixture with fs$_0$= 39% which yields fs$_s$= 38% reproduced well our previously reported data for the same mixture (fs$_s$= 36%).[7]

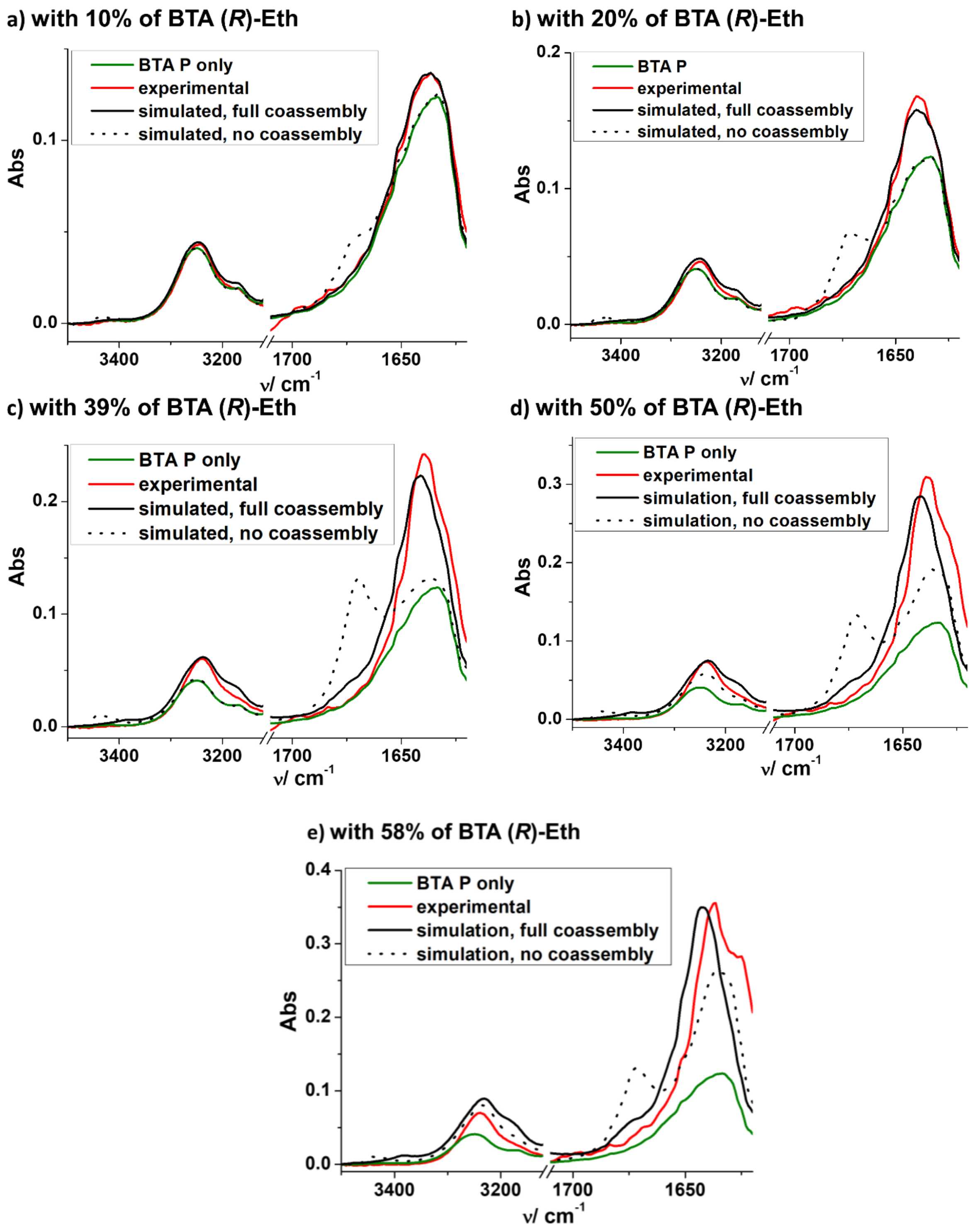

Figure S11. FT-IR analyses of the "sergeants-and-soldiers"-type mixtures containing **BTA P** (5.8 mM) and **BTA (*R*)-Eth** (0.65 mM─8.0 mM, 10%≤ fs_0≤ 58%) in toluene at 293 K. Zoom on the N─H and C=O regions. The FT-IR spectrum for **BTA P** only (5.8 mM) is also shown. FT-IR spectra for full and no coassembly have been simulated as indicated in the General Methods. For the attribution of the bands, see Figure S9.

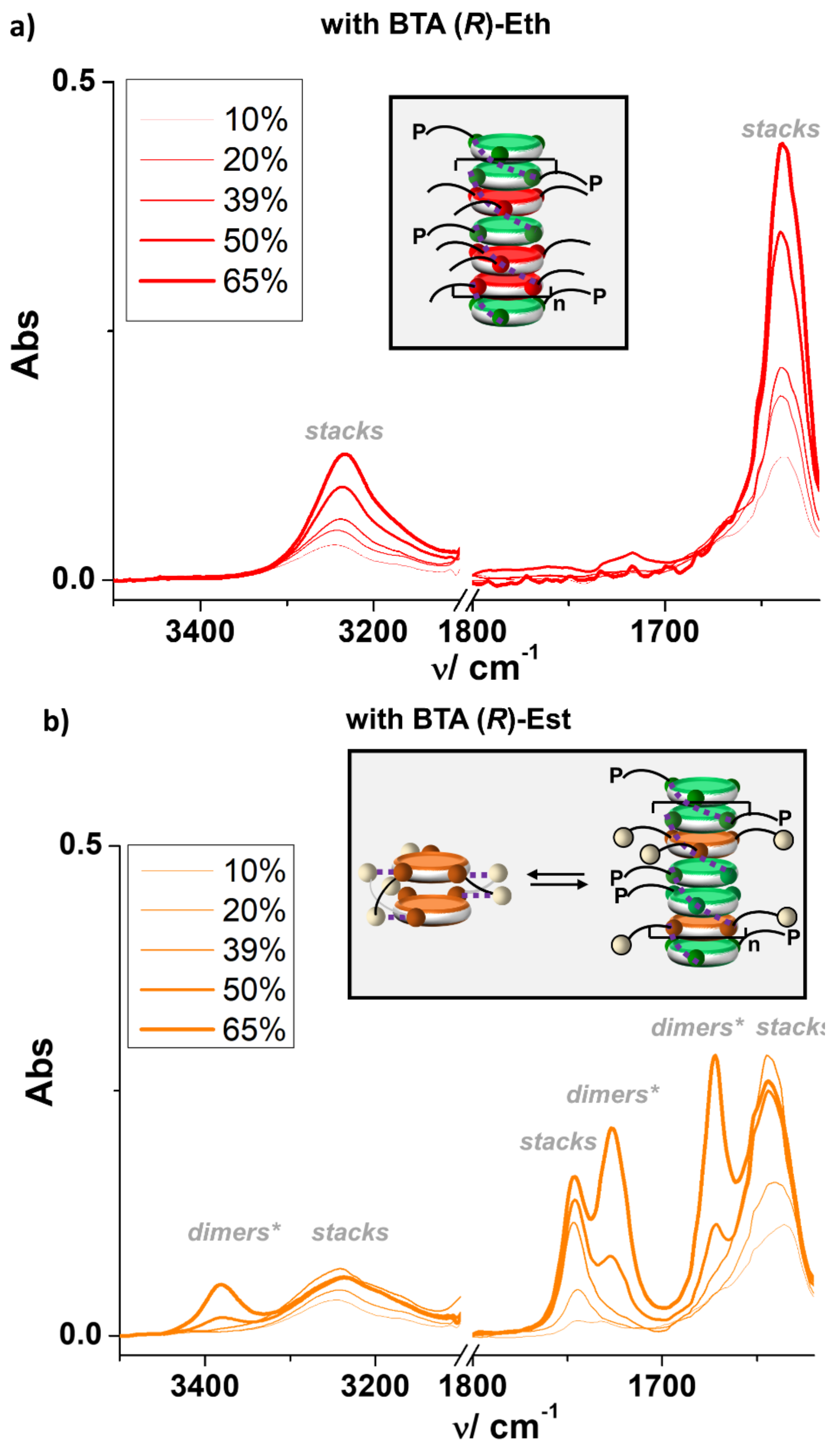

Figure S12. FT-IR analyses of the “sergeants-and-soldiers”-type mixtures containing **BTA P** coordinated to $[Cu(OAc)_2]$ (**BTA P**/[Cu]= 4, [**BTA P**]= 5.8 mM) and either **BTA (*R*)-Eth** (0.65 mM—10.8 mM, 10%≤ fs_0≤ 65%) or **BTA (*R*)-Est** (0.65 mM—10.8 mM, 10%≤ fs_0≤ 65%) in toluene at 293 K. Zoom on the N—H and C=O regions. Dimers*= these bands can also include mixed species between **BTA P** and **BTA (*R*)-Est** (not stacks) for which N—H can be bonded to ester and amide C=O.[2,6]

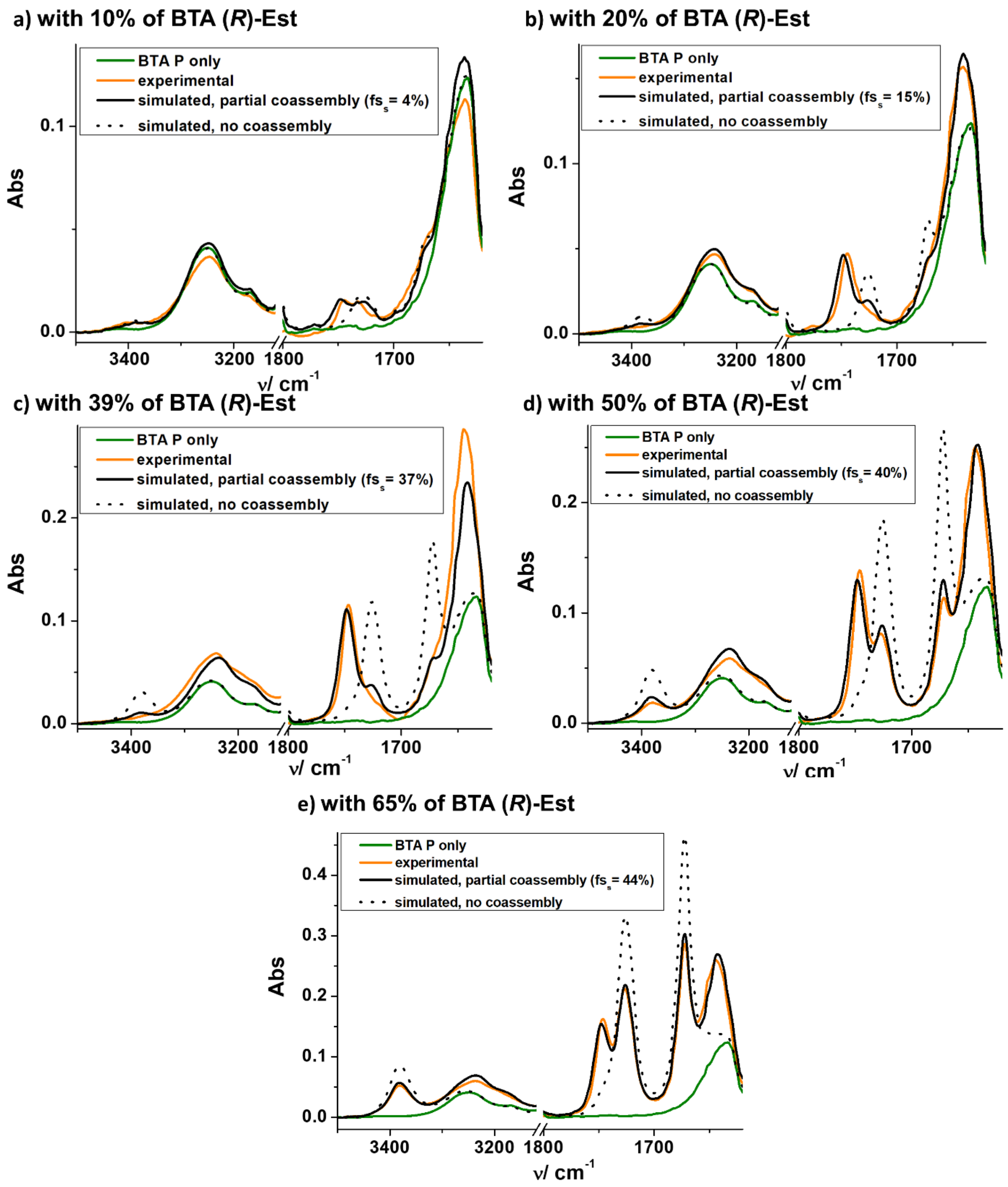

Figure S13. FT-IR analyses of the "sergeants-and-soldiers"-type mixtures containing **BTA P** coordinated to [Cu(OAc)$_2$] (**BTA P**/[Cu]= 4, [**BTA P**]= 5.8 mM) and **BTA (*R*)-Est** (0.65 mM─10.8 mM, 10%≤ fs_0≤ 65%) in toluene at 293 K. Zoom on the N─H and C=O regions. The FT-IR spectrum for **BTA P** only (5.8 mM) is also shown. FT-IR spectra for full and no coassembly have been simulated as indicated in the General Methods. For the attribution of the bands, see Figure S8. Experimental spectrum for the mixture with fs_0= 39% which yields fs_s= 37% reproduced well our previously reported data for the same mixture (fs_s= 36%).[7]

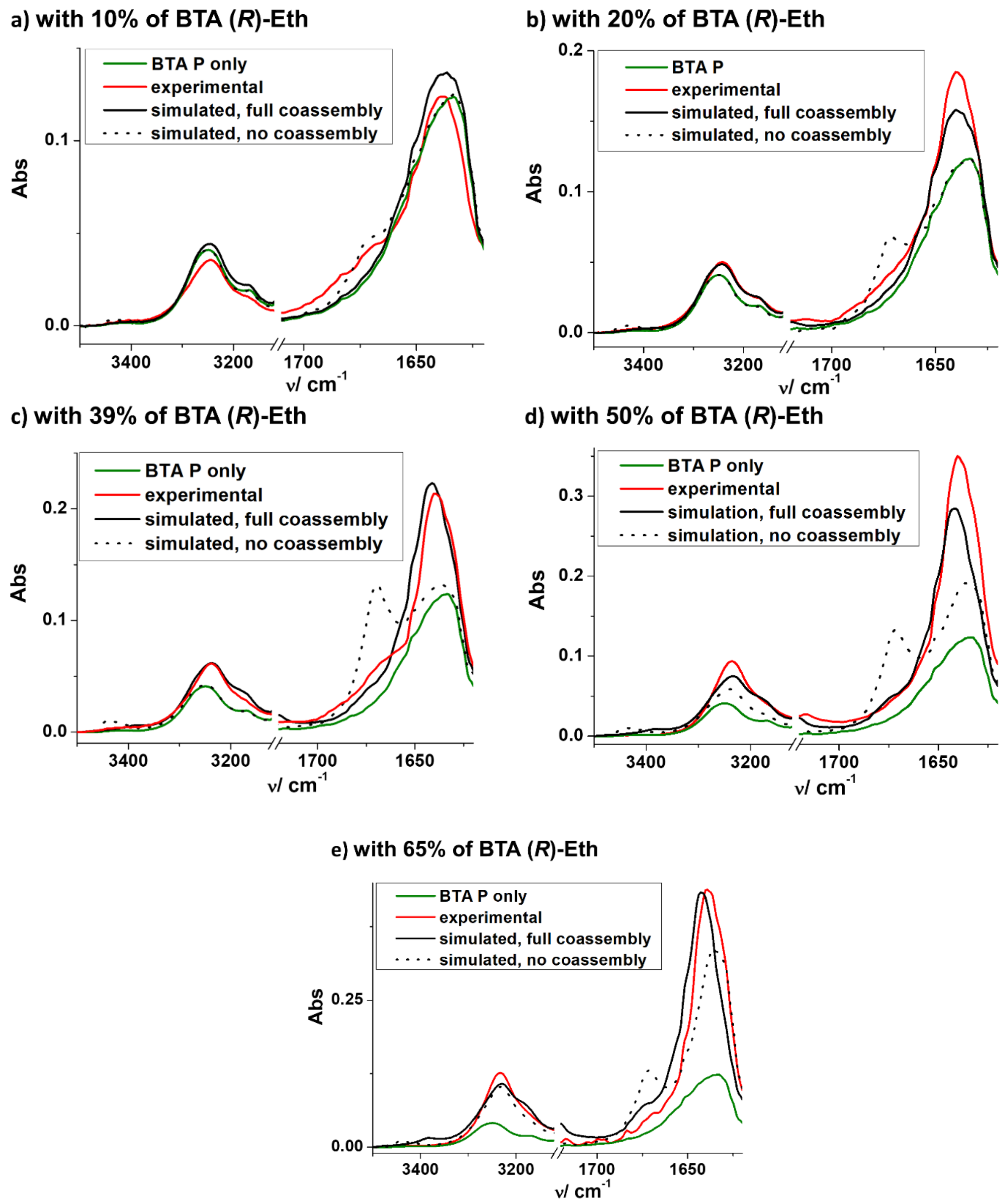

Figure S14. FT-IR analyses of the "sergeants-and-soldiers"-type mixtures containing **BTA P** coordinated to [Cu(OAc)$_2$] (**BTA P**/[Cu]= 4, [**BTA P**]= 5.8 mM) and **BTA (*R*)-Eth** (0.65 mM─10.8 mM, 10%≤ fs_0≤ 65%) in toluene at 293 K. Zoom on the N─H and C=O regions. The FT-IR spectrum for **BTA P** only (5.8 mM) is also shown. FT-IR spectra for full and no coassembly have been simulated as indicated in the General Methods. For the attribution of the bands, see Figure S8.

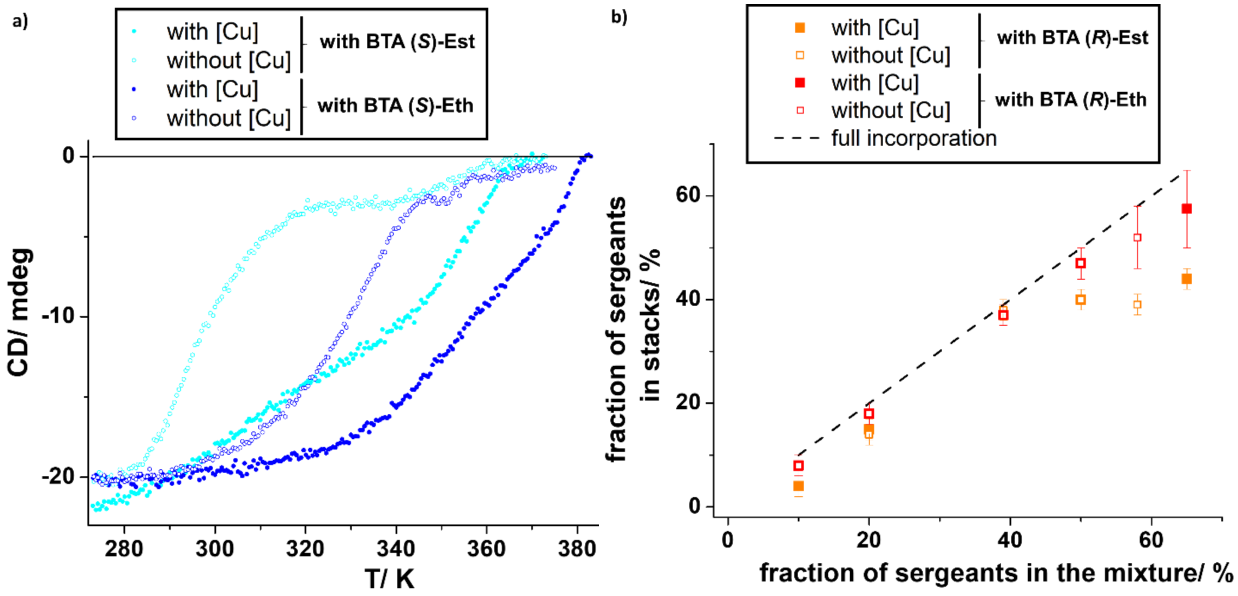

Figure S15. a) CD curves (λ= 295 nm) as a function of the temperature for the mixtures containing **BTA P** (5.8 mM, with and without copper, **BTA P**/[Cu]= 4 when copper present) and either **BTA (*S*)-Eth** or **BTA (*S*)-Est** (5.8 mM). Data recorded upon heating (0.5 K.min^{-1}). b) Plot of the fraction of "sergeant" in the stacks (fs_s) as a function of the fraction of "sergeant" initially introduced into the mixtures (fs_0) for the "sergeants-and-soldiers"-type mixtures (10%≤ fs_0≤ 65%) with and without [Cu(OAc)$_2$] coordinated to **BTA P**. Most of the data points with and without copper overlap.

Interpretation: the presence of [Cu(OAc)$_2$] increases the stability of the coassemblies for both **BTA (*S*)-Eth and BTA (*S*)-Est** but coassemblies of the former are still significantly more stable. The presence of [Cu(OAc)$_2$] has no significant influence on the amount of "sergeants" intercalated with **BTA P**: BTA Eth intercalated more efficiently into **BTA P** than BTA Est whether copper is coordinated to **BTA P** or not. The increased stability in presence of copper may be attributed to his ability to "mask" the hydrogen-bond competitive nature of the PPh_2 moiety.[8] **BTA (*S*)-Eth** and **BTA (*S*)-Est** intercalate more efficiently into **BTA P** that our previously-investigated "sergeant" derived from cyclohexylalanine.[7]

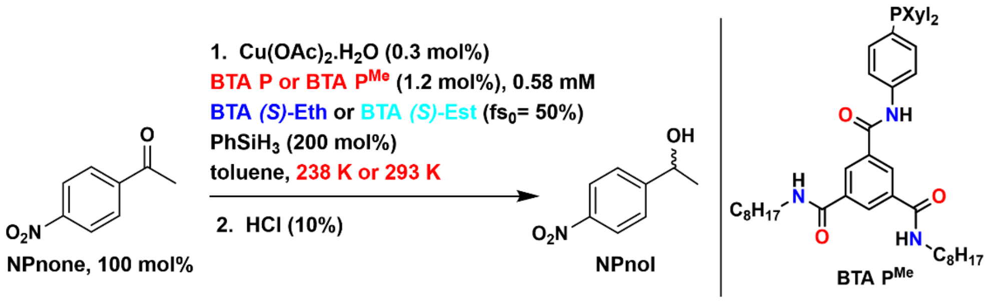

entry	BTA ligand	« sergent »	T (K)	conversion (%)	|ee| (%)
1	**BTA P**	**BTA (*S*)-Eth**	293	97	51
2	**BTA P**	**BTA (*S*)-Est**	293	96	41
3	**BTA P^{Me}**	**BTA (*S*)-Eth**	238	89	78
4	**BTA P^{Me}**	**BTA (*S*)-Est**	238	30	69

Figure S16. Compared catalytic performances for the two "sergeants" with different BTA ligands (**BTA P** or **BTA P^{Me}**) and at different temperatures (293 K or 238 K). **BTA P^{Me}** was previously shown to exhibit higher enantioselectivities than **BTA P**.[9]

The highest ee (highlighted in light orange) was obtained by combining **BTA P^{Me}** and **BTA (*S*)-Eth** at 238 K. At this temperature, **BTA (*S*)-Eth** outperforms **BTA (*S*)-Est** both in terms **of** activity and selectivity.

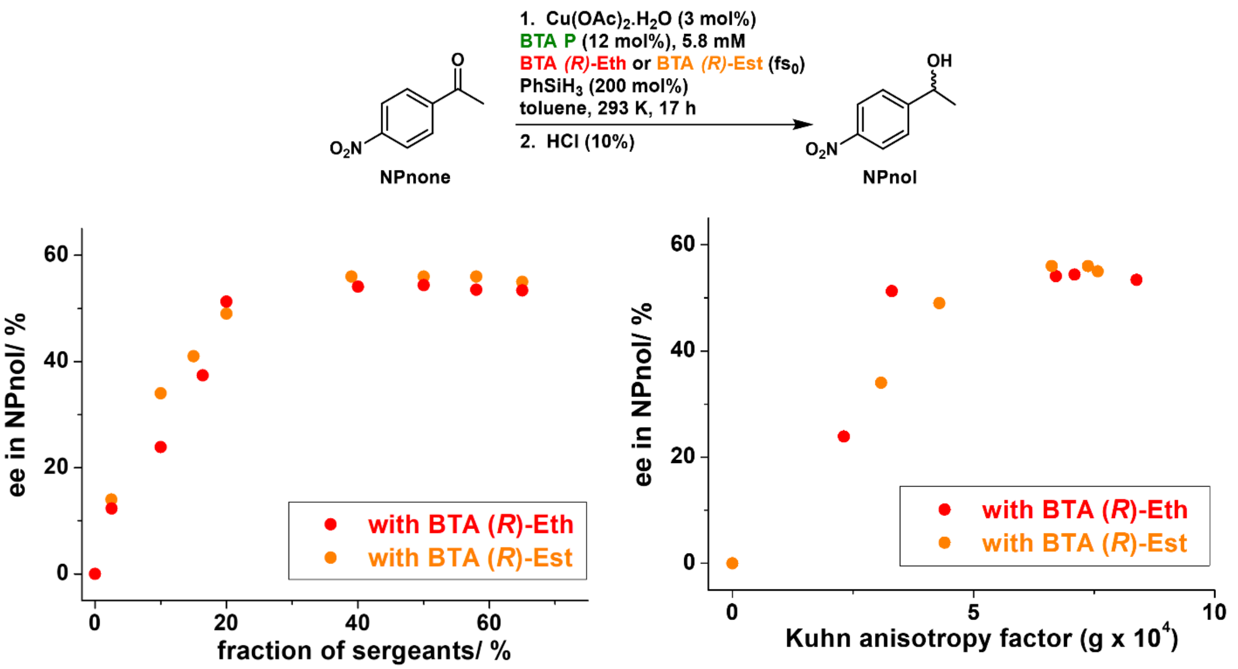

Figure S17. a) Enantiomeric excess (ee) in NPnol as a function of the fraction of "sergeants" (either **BTA (*R*)-Eth** or **BTA (*R*)-Est**) in the helical catalysts under our classical conditions. Conversion > 99% in all cases. The concentration in **BTA P** is set constant to 5.8 mM thus the total BTA concentration for each mixture is largely above the critical concentration for the formation of stable helical coassemblies/copolymers. Data for the **BTA (*R*)-Est** containing mixtures correspond to those reported previously by our group.[7] b) Plot of the enantiomeric excess (ee) in **NPnol** as a function of the Kuhn anisotropy factor (g) for both types of coassemblies. The g values are extracted from the CD analyses of each mixture (Figure S12). The good correlation between the ee in **NPnol** and the optical purity of the supramolecular helical catalyst (reflected by the g value) is consistent with our previous result.[7]

Interpretation: **BTA (*R*)-Eth** and **BTA (*R*)-Est** helical catalysts exhibit similar selectivity under these conditions.

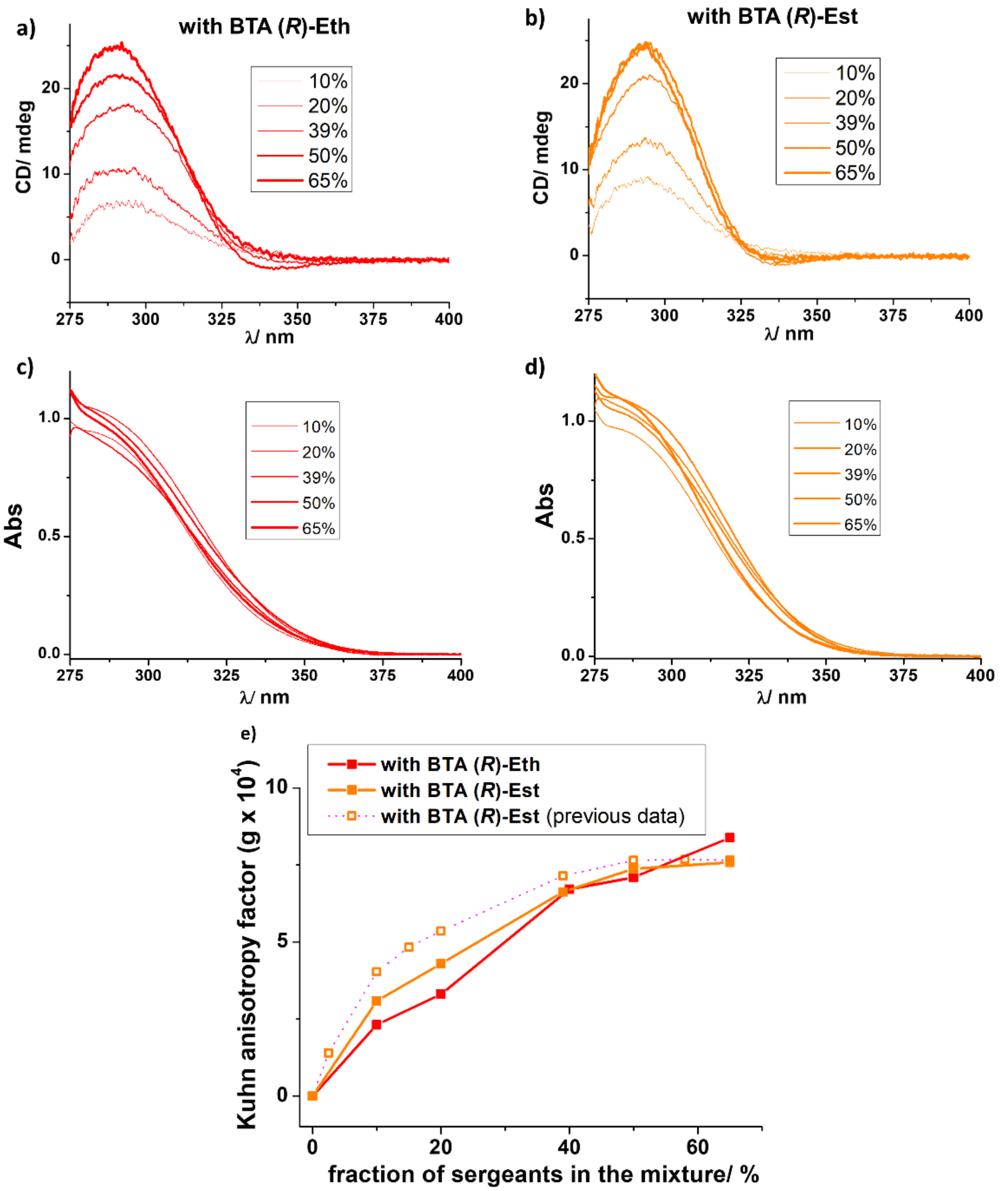

Figure S18. CD and UV-Vis analyses of the helical coassemblies containing **BTA P** coordinated to [Cu(OAc)$_2$] (**BTA P**/[Cu]= 4, [**BTA P**]= 5.8 mM) and either **BTA (*R*)-Eth** (0.65 mM—10.8 mM, 10%≤ fs$_0$≤ 65%, a-c) or **BTA (*R*)-Est** (0.65 mM—10.8 mM, 10%≤ fs$_0$≤ 65%, b-d) in toluene at 293 K under our classical conditions. Only **BTA P** absorbs in this region and thus the observed CD signal is an induced CD signal.[10] Variation in the UV-Vis intensity is mainly related to the transition between single (at high fraction of "sergeant") and crosslinked helices (at low fraction of "sergeant"), with copper acting as crosslinks.[8] e) Plot of the Kuhn anisotropy factor (g) as a function of the fraction of "sergeant" for both types of coassemblies. The Kuhn anisotropy factor is determined as $g=\theta^{295}/(32982\times Abs^{295})$ where θ^{295} and Abs^{295} are the ellipticity and UV/Vis absorbance measured at λ=295 nm, respectively. For **BTA (*R*)-Est** containing mixtures, **s**light variation between these data and those previously reported[8] probably comes from the fact that crosslinked helices are present at intermediate fraction values.

Interpretation: **BTA (*R*)-Eth** and **BTA (*R*)-Est** helical coassemblies exhibit similar chiroptical properties under these conditions.

General methods

Synthetic procedures: The syntheses of **BTA P**,[11] **BTA P^{Me}**,[9] **BTA (*S*)-Est**,[12] and **BTA (*R*)-Est**[7] were reported previously by our group, and **BTA C8**[13] was prepared according to the literature. The synthesis of **BTA (*S*)-Eth** and **BTA (*R*)-Eth** is described below. (*R*)-Leucinol (99.5%, BLD Pharm), (*S*)-Leucinol (96%, TCI Europe), phthalic anhydride (99%, Alfa Aesar), NaH (60% dispersion in mineral oil, TCI Europe), bromododecane, (98%, TCI Europe), hydrazine monohydrate (> 99%, Alfa Aesar), benzene-1,3,5-tricarbonyl trichloride (98%, Alfa Aesar), 1-(4-nitrophenyl)ethanone (>98%, Alfa Aesar), $PhSiH_3$ (>97%, Alfa Aesar) and $[Cu(OAc)_2{\cdot}H_2O]$ were purchased and used without any purification. Deuterated solvents were purchased from Eurisotop and used without further purification. Anhydrous solvents were obtained from a solvent purification system (IT-Inc). Purification by flash chromatography was performed by adsorbing the samples on silica; the adsorbed samples were introduced into the solid loader and purified by means of a Reveleris X2 purification system (Buchi®) using pre-packed silica cartridges Ecoflex® (irregular 50 µm silica) of 40 g, 80 g or 120 g. ^{1}H NMR spectra were recorded on a Brucker 400 Avance or 300 Avance (400 and 300 MHz, respectively) and are calibrated with residual $CDCl_3$ and acetone-*d*6 protons signals at δ=7.26 ppm and δ= 2.05 ppm, respectively. ^{13}C NMR spectra were recorded on a Brucker 400 Avance or 300 Avance (100 and 75 MHz, respectively) and are calibrated with the acetone-*d*6 signal at δ= 29.84 ppm. Data are reported as follows: chemical shift (δ ppm), multiplicity (s= singlet, br s= broad singlet, d= doublet, t= triplet, m= multiplet), coupling constant (Hz) and integration. Exact mass measurements (HRMS) were obtained on a TQ R30-10 HRMS spectrometer by ESI^+ ionization and are reported in *m/z* for the major signal. FT-IR analysis was performed on a Bruker Tensor 27 spectrometer in ATR (diamond probe).

Chiral HPLC analyses: The optical purity of **BTA (*S*)-Eth** (ee> 99%, de> 93%) and **BTA (*R*)-Eth** (ee> 99%, de> 99%) was controlled by analytical HPLC ((*S*,*S*)Whelk-O1, heptane/isopropanol (90/10) as mobile phase, 1 mL/min). See Figure S24.

Circular Dichroism (CD) analyses: Circular dichroism (CD) measurements were performed on a Jasco J-1500 spectrometer equipped with a Peltier thermostated cell holder and Xe laser. <u>CD spectra of Figure S2</u>: they were recorded at 293 K with the following parameters: 50 $nm.min^{-1}$ sweep rate, 0.05 nm data pitch, 2.0 nm bandwidth, and between 350 and 210 nm. The solutions were placed into a cylindrical spectrosil quartz cell of 0.05 mm pathlength (Starna® 31/Q/0.05). Methylcyclohexane and cell contributions at the same temperature were subtracted from the obtained signals. The CD intensity is reported as the molar extinction coefficient, calculated as $\Delta\varepsilon = \theta/(32982\times[\textbf{BTA P}]\times l)$, with θ= ellipticity (in mdeg), [**BTA P**]= concentration in **BTA P** (in $mol.L^{-1}$), and l= cell pathlength (in cm). <u>CD analyses of Figures 2b and S15a</u>: solutions were placed into a cylindrical spectrosil quartz cells of 0.10 mm pathlength (Starna® 31/Q/0.10), and the ellipticity and UV Abs were recorded at λ= 295 nm during a heating process (0.5 $K.min^{-1}$). Variable-temperature CD analyses were conducted between 273 K and 373 K or 383 K, and CD and UV data were recorded at 295 nm every 0.5 K. <u>CD analyses of Figures 3c and S18</u>: CD spectra

were recorded at 293 K with the following parameters: 50 nm.min^{-1} sweep rate, 0.05 nm data pitch, 2.0 nm bandwidth, and between 400 and 275 nm with solutions placed into a cylindrical spectrosil quartz cells of 0.10 mm pathlength (Starna® 31/Q/0.10). The pathlength of the cell was adapted for the most diluted solutions and the CD intensity is reported as the molar extinction coefficient. Toluene and cell contributions at the same temperature were subtracted from the obtained signals. The Kuhn anisotropy factor is determined as $g=\theta^{295}/(32982\times Abs^{295})$ where θ^{295} and Abs^{295} are the ellipticity and UV/Vis absorbance measured at λ=295 nm, respectively.
All solutions were pre-heated before measurements. For all samples, the LD contribution was negligible (ΔLD < 0.005 dOD) and the shape of the CD signal was independent of the orientation of the quartz cells.

UV-Vis analyses: UV-Vis absorption spectra were extracted from CD on each of the above samples and obtained after correction of the absorption of air, solvent, and cell at 293 K.

Fourier-Transform Infrared (FT-IR) analyses: FTIR measurements were performed on a Nicolet iS10 spectrometer. Spectra of solutions in toluene and methylcyclohexane were measured in 0.02 cm pathlength CaF_2 cells at room temperature and were corrected for air, solvent and cell absorption. Spectra for homoassemblies in Figures S1 and S2 were plotted as the molar extinction coefficient, ε, defined as ε= Abs/([BTA]×l) with [BTA] and l the total concentration in BTA monomers (in mol.L^{-1}) and the cell pathlength (in cm), respectively. Procedure for simulated FT-IR spectra for solutions with BTA Est (Figures S10 and S13):[8] As no free N─ H can be detected in the FTIR spectra of these mixtures (Figure S9b and 12b), the influence of the stack ends is neglected. The concentration of dimers is extracted by fitting the ester carbonyl region of the experimental FT-IR spectrum (1700–1800 cm^{-1}) with the individual spectra of **BTA (*R*)-Est** (representative of dimers, bonded ester C=O, ν≈1725 cm^{-1}) and of BTA Met (used as blueprint of **BTA (*R*)-Est** monomers in stacks, free ester C=O, ν ≈1745 cm^{-1}).[1,2] The fraction of **BTA (*R*)-Est** in stacks (fs_s) is thus deduced from the concentration of remaining dimers. The extracted fs_s values are plotted as a function of fs_0 in Figures 2d (without copper) and Figure S15b (both with and without copper). The simulated spectrum for segregation of the two partners in their own assemblies (no coassembly) is built by summing their individual FT-IR spectra, **BTA (*R*)-Est** (0.65 mM─10.8 mM) and **BTA P** (5.8 mM). The simulated spectrum for partial coassembly is built by summing the FT-IR spectra of the remaining **BTA (*R*)-Est** dimers, of **BTA P**, and of BTA Met (used as blueprint of **BTA (*R*)-Est** monomers in stacks). Procedure for simulated FT-IR spectra for solutions with BTA Eth (Figures 2c, S11 and S14): As no free N─ H can be detected in the FTIR spectra of these mixtures (Figure S9a and S12a), the influence of the stack ends is neglected. We only consider the presence of stacks and monomers for these mixtures even though competing species cannot be discarded. The simulated spectrum for segregation of the two partners in their own assemblies (no coassembly) is built by summing their individual FT-IR spectra, **BTA (*R*)-Eth** (0.65 mM─10.8 mM) and **BTA P** (5.8 mM). The simulated spectrum for full coassembly is built by summing the FT-IR spectra of the remaining of **BTA P** and of BTA Met (used as blueprint of **BTA (*R*)-Eth** monomers in stacks). The good fit between experimental and simulated spectra

is consistent with full coassembly for the “sergeant” ratio. However, for the mixtures containing 50% and 65% of **BTA (*R*)-Eth,** a fraction of **BTA (*R*)-Eth** forms stacks on their own (as deduced from Figure S1a) and thus homo stacks of **BTA (*R*)-Eth** and mixed stacks of **BTA P** and **BTA (*R*)-Eth** cannot be formally distinguished. We thus assign an error bar value for these mixtures in Figures 2d and S15b (fs_s= 47±3% and fs_s= 57.5±7.5% for fs_0= 50% and fs_0= 65%, respectively) whilst for the other mixtures we took fs_s= fs_0.

Isothermal Titration Calorimetry (ITC) analyses: ITC data were recorded on a Microcal VP-ITC apparatus at the desired temperature, injecting a toluene solution containing one BTA monomer (Figure S5) or a mixture of BTA monomers (Figures 2a, 4 and S7) into neat toluene. Injections of 5 µL over 10 seconds were performed every 480 seconds at a stirring rate of 300 rpm.

Nuclear Magnetic Resonance (NMR) analyses: ^{1}H NMR spectra (Figure S3) were recorded on a Bruker Avance III 600 spectrometer (14.1 T) operating at a ^{1}H Larmor frequency of 600.4 MHz. The spectrometer is equipped with a 5 mm Observe broadband BBFO probe (^{15}N-^{31}P/^{19}F/^{1}H) with the z-axis gradient coil maximum gradient strength of 55.35 G/cm.
The ^{1}H NMR spectra were recorded using a pulse sequence of proton with a spectral width of 9615.4 Hz an acquisition time of 3.4 s, and a relaxation delay of 1 s. Calibration was done on the residual solvent peak of C_7D_8 (^{1}H: 2.09 ppm).
DOSY experiments were performed using stimulated echo and longitudinal eddy delay with bipolar gradients and two spoil gradients (ledbpgp2s). The diffusion time was Δ= 0.05 s. The duration of the magnetic field pulse gradients $\delta/2$ for the samples containing **BTA Eth** was adjusted to 1600 µs (5.8 mM and 11.6 mM) or 1700 µs (30.5 mM). For the samples containing **BTA Est**, the duration of the magnetic field pulse gradients $\delta/2$ was adjusted to 1150 (5.8 mM), 1450 (11.6 mM), or 1650 µs (30.5 mM). The delay for gradient recovery was of 0.1 ms and the eddy current delay was of 5 ms. For each DOSY-NMR experiment, a series of 16 spectra on 32 K data points were collected. The pulse gradients (g) were incremented from 2 to 98% of the maximum gradient strength in a linear ramp with a total experiment time of 55 min. The temperature was set and controlled to 300 K with an air flow of 270 L.h^{-1} in order to avoid any temperature fluctuations due to sample heating during the magnetic field pulse gradients. After Fourier transformation and baseline correction, the diffusion dimension was processed with the Topspin 3.6.1 software. Diffusion coefficients, processed with a line broadening of 1 Hz, were calculated by Gaussian fits with Dynamics Center (Bruker software).

Viscosimetry: viscosimetry measurements were performed with a Cannon-Manning Semi-Micro U-tube viscosimeter (size 100) immersed in a thermostated water bath (293 K). Viscosity values are reported as an average of 3 measurements and normalized by that of toluene measured at the same temperature.

Pseudo-phase diagrams (Figure 2e-1f): Data extracted from ITC (293 K and 333 K) and CD (5.8 mM) analyses. For CD, points are taken at the rising of the CD signal (i.e., elongation temperature, T_e) and 80% of the plateau of the CD signal. It yields the following values: For 1:1 coassemblies

with **BTA (*R*)-Eth:** monomers≤ 0.04 mM≤ monomers + stacks≤ 0.3 mM≤ stacks (ITC, T= 293 K). monomers≤ 0.76 mM≤ monomers + stacks≤ 1.72 mM≤ stacks (ITC, T= 323 K). stacks≤ 323 K≤ short stacks + competing species≤ 372 K≤ monomers (CD, c= 11.6 mM). For 1:1 coassemblies with **BTA (*R*)-Est:** monomers + dimers≤ 0.4 mM≤ competing species≤ 1.4 mM≤ stacks (ITC, T= 293 K). stacks≤ 293 K≤ competing species≤ 364 K≤ monomers + dimers (CD, c= 11.6 mM).

Preparation of FT-IR and CD solutions and solutions for catalytic experiments: see below

Catalytic experiments, Tables S3-S6

With BTA P= 5.8 mM (Figure S11): Experiments with BTA Eth mixtures were performed similarly to our previous study with BTA Est mixtures.[7] A pre-catalytic mixture composed of the ligand, the copper salt and the substrate was prepared as follows: oven-dried test tubes were loaded with a stock solution prepared by mixing **BTA P** and [$Cu(OAc)_2 \cdot H_2O$], divided in order to get **BTA P** (2.4 mg, 3.5 µmol, 12.0 mol%) and [$Cu(OAc)_2 \cdot H_2O$] (0.17 mg, 0.87 µmol, 3.0 mol%) in dry THF (500 µL) in each tube. The solvent was removed under vacuum and the tubes were kept under vacuum (10^{-3} mbar) for 1 hour. Then 1-(4-nitrophenyl)ethanone (**NPnone**, 4.8 mg, 0.029 mmol) was added.

Preparation of the solutions of sergeants: A 17.4 mM stock solution of the **BTA (*R*)-Eth** was prepared in dry toluene as well as a 1.74 mM stock solution by dilution. Then the desired amount of **BTA (*R*)-Eth** stock solution was added to each vial as follows: 45 mg of the 1.74 mM stock solution (0.09 µmol, 0.15 mM, fs_0= 2.5%), 193 mg of the 1.74 mM stock solution (0.39 µmol, 0.65 mM, fs_0= 10%), 31 mg of the 17.4 mM stock solution (0.66 µmol, 1.1 mM, fs_0= 16.4%), 44 mg of the 17.4 mM stock solution (0.87 µmol, 1.5 mM, fs_0= 20%), 111 mg of the 17.4 mM stock solution (2.34 µmol, 3.9 mM, fs_0= 40%), 240 mg of the 17.4 mM stock solution (4.81 µmol, 8.0 mM, fs_0= 58%), and 322 mg of the 17.4 mM stock solution (6.46 µmol, 10.8 mM, fs_0= 65%). Finally, dry toluene was added to each vial in order to get a total volume of 600 µL. The mixtures were stirred for 15 min at 293 K and then briefly heated up to the solvent boiling point (≈ 383 K). After cooling to room temperature, $PhSiH_3$ (8.0 µL, 0.058 mmol) was added to each test tube and the mixtures were stirred for 17 hours.

Typical work-up: Aqueous solution of HCl (10 wt%, 500 µL) was added and the mixtures were stirred for 30 min (until the solution became transparent). Then, the products were extracted with Et_2O (500 µL) and AcOEt (500 µL) and the organic phase was passed through a small silica plug. The solvents were evaporated and the crude material was analyzed by NMR and by chiral GC. Conversion and enantiomeric excess (ee) were determined by chiral GC analysis. Ee are indicated as positive and negative when **(*S*)-NPnol** and **(*R*)-NPnol** are the major enantiomers, respectively. Composition of the catalytic mixtures, g values of the coassemblies, and catalytic performance (conversion, ee) provided by the catalytic mixtures are compiled in Tables S3-S4.

Chiral GC analyses: The optical purity was determined by GC analysis: Chiral Cyclosil-B column, 30 m × 250 µm x 0.25 µm, inlet pressure= 12.6 psi. Injection temperature= 250°C; detector temperature= 300°C; column temperature= 135°C. Retention time: 18 min (**NPnone**), 50 min ((*R*)-enantiomer of **NPnol**), 53 min ((*S*)-enantiomer of **NPnol**).

With BTA P= 0.58 mM (Figure 2b): A pre-catalytic mixture composed of the ligand, the copper salt and the substrate was prepared as follows: oven-dried test tubes were loaded with a stock solution prepared by mixing **BTA P** and [$Cu(OAc)_2 \cdot H_2O$], divided in order to get **BTA P** (0.24 mg, 0.35 µmol, 1.2 mol%) and [$Cu(OAc)_2 \cdot H_2O$] (0.017 mg, 0.087 µmol, 0.3 mol%) in dry THF (500 µL) in each tube. The solvent was removed under vacuum and the tubes were kept under

vacuum (10^{-3} mbar) for 1 hour. Then 1-(4-nitrophenyl)ethanone (**NPnone**, 4.8 mg, 0.029 mmol) was added.

Preparation of the solutions of sergeants: A 1.74 mM stock solution of the **BTA (*R*)-Est** or **BTA (*R*)-Eth** was prepared in dry toluene as well as a 0.17 mM stock solution by dilution. Then the desired amount of **BTA (*R*)-Est** or **BTA (*R*)-Eth** stock solution was added to each vial as follows: 45 mg of the 0.17 mM stock solution (0.009 µmol, 0.015 mM, fs_0= 2.5%), 90 mg of the 0.17 mM stock solution (0.018 µmol, 0.03 mM, fs_0= 5%), 193 mg of the 0.17 mM stock solution (0.039 µmol, 0.065 mM, fs_0= 10%), 31 mg of the 1.74 mM stock solution (0.061 µmol, 0.10 mM, fs_0= 15%), 44 mg of the 1.74 mM stock solution (0.087 µmol, 0.15 mM, fs_0= 20%), 111 mg of the 1.74 mM stock solution (0.23 µmol, 0.39 mM, fs_0= 40%) and 174 mg of the 1.74 mM stock solution (0.35 µmol, 0.58 mM, fs_0= 50%).. Composition of the catalytic mixtures, g values of the coassemblies, and catalytic performance (conversion, enantioselectivity) provided by the catalytic mixtures are compiled in Tables S5-S6.

With BTA P^{Me}= 0.58 mM (Figure S16): A pre-catalytic mixture composed of the ligand, the copper salt and the substrate was prepared as follows: oven-dried test tubes were loaded with a stock solution prepared by mixing **BTA P^{Me}** and [$Cu(OAc)_2 \cdot H_2O$], divided in order to get **BTA P^{Me}** (0.24 mg, 0.35 µmol, 1.2 mol%) and [$Cu(OAc)_2 \cdot H_2O$] (0.017 mg, 0.087 µmol, 0.3 mol%) in dry THF (500 µL) in each tube. The solvent was removed under vacuum and the tubes were kept under vacuum (10^{-3} mbar) for 1 hour. Then 1-(4-nitrophenyl)ethanone (**NPnone**, 4.8 mg, 0.029 mmol) was added as well as 174 mg of a 1.74 mM stock solution of **BTA (*R*)-Eth** or **BTA (*R*)-Est** (0.35 µmol, 0.58 mM, fs_0= 50%) in toluene. Finally, dry toluene was added to each vial in order to get a total volume of 600 µL. The mixtures were stirred for 15 min at 293 K and then briefly heated up to the solvent boiling point (≈ 383 K). After cooling to 238 K, $PhSiH_3$ (8.0 µL, 0.058 mmol) was added to each test tube and the mixtures were stirred for 40 hours. Work-up and GC analyses were performed as indicated above. Catalytic results are provided in Figure S16.

Representative chiral GC analyses are given in pages S36-S42.

Table S3 Composition, enantioselectivity and g values for each mixture composed **of BTA P** and **BTA (*R*)-Est**. **BTA P**= 5.8 mM.

Entry	[“**sergeant**”] (mM)	f_{S0} (%)	ee±1% in NPnol (%)[7]	g±0.2 (anisotropy factor, ×10^4)[7]	g±0.2 (anisotropy factor, ×10^4) [*reproduced data]*
1	0.15, (*R*)	2.5	+14	1.4	*na*
2	0.64, (*R*)	10	+34	4.0	2.3
3	1.0, (*R*)	15	+41	4.8	*na*
4	1.5, (*R*)	20	+49	5.4	3.3
5	3.7, (*R*)	39	+56	7.1	6.7
6	5.8, (*R*)	50	+56	7.6	7.1
7	8.0, (*R*)	58	+56	7.7	*na*
8	10.8, (*R*)	65	+55	7.7	8.4

na: not analyzed. Conversion> 99%.

Table S4 Composition, enantioselectivity and g values for each mixture composed **of BTA P** and **BTA (*R*)-Eth**. **BTA P**= 5.8 mM.

Entry	[“**sergeant**”] (mM)	f_{S0} (%)	ee±1% in NPnol (%)	g±0.2 (anisotropy factor, ×10^4)
1	0.15, (*R*)	2.5	+12	*na*
2	0.64, (*R*)	10	+24	2.3
3	1.1, (*R*)	16.4	+37	*na*
4	1.5, (*R*)	20	+51	3.3
5	3.9, (*R*)	40	+54	6.7
6	5.8, (*R*)	50	+54	7.1
7	8.0, (*R*)	58	+54	*na*
8	10.8, (*R*)	65	+53	8.4

na: not analyzed. Conversion> 99%.

Table S5 Composition and enantioselectivity for each mixture composed **of BTA P** and **BTA (*S*)-Est**. **BTA P**= 0.58 mM.

Entry	[**"sergeant"**] (mM)	f_{S0} (%)	conversion (%)	ee±1% in NPnol (%)
1	0.02, (*S*)	2.5	86	0
2	0.03, (*S*)	5	86	−15
3	0.065, (*S*)	10	95	−22
4	0.15, (*S*)	20	97	−26
5	0.39, (*S*)	40	96	−36
6	0.58, (*S*)	50	96	−41

Table S6 Composition and enantioselectivity for each mixture composed **of BTA P** and **BTA (*S*)-Eth**. **BTA P**= 0.58 mM.

Entry	[**"sergeant"**] (mM)	f_{S0} (%)	conversion (%)	ee±1% in NPnol (%)
1	0.02, (*S*)	2.5	86	0
2	0.03, (*S*)	5	85	−17
3	0.065, (*S*)	10	90	−35
4	0.1, (*S*)	15	97	−37
5	0.15, (*S*)	20	97	−42
6	0.39, (*S*)	40	95	−49
7	0.58, (*S*)	50	97	−51

Solutions for ITC, NMR, FT-IR, CD and rheology analyses

Homoassemblies (Figures S1-S5): a desired amount of the BTA monomer was mixed with toluene, C_7D_8 or MCH, the mixture was stirred at room temperature and then briefly heated up to the solvent boiling point. After cooling to room temperature, the solutions were transferred to a NMR tube, to a quartz cell, or to a CaF_2 cell and analyzed rapidly.

Coassemblies without copper (Figures 2b, 2c, 3a, 4, S7, S9, S10, S11): The desired amounts of "soldier" (**BTA P** or **BTA C8**) and "sergeants" (BTA Eth or BTA Est) were loaded in oven-dried test tubes and dry toluene was added to each vial in order to get a total volume of 600 μL. The mixtures were stirred for 15 min at 293 K and then briefly heated up to the solvent boiling point ($\approx$ 383 K). After cooling to room temperature, the solutions were transferred to a quartz cell, a CaF_2 cell, ITC cell or U-tube viscosimeter and analyzed rapidly. For rheology at various concentrations (Figure 3a), solutions were obtained by dilution of the more concentrated one.

Coassemblies with copper (Figures 3c, S12, S13, S14, S15, S18): A mixture composed of the ligand and the copper salt was prepared as follows: oven-dried test tubes were loaded with a stock solution prepared by mixing **BTA P** and [$Cu(OAc)_2 \cdot H_2O$], divided in order to get **BTA P** (2.4 mg, 3.5 μmol) and [$Cu(OAc)_2 \cdot H_2O$] (0.17 mg, 0.87 μmol) in dry THF (500 μL) in each tube. The solvent was removed under vacuum and the tubes were kept under vacuum (10^{-3} mbar) for 1 hour. The desired amount of "sergeant" (BTA Eth or BTA Est) was added to the solid (by means of stock solutions mentioned in the part dealing with preparation of the catalytic mixtures). Dry toluene was added to each vial in order to get a total volume of 600 μL. The mixtures were stirred for 15 min at 293 K and then briefly heated up to the solvent boiling point ($\approx$ 383 K). After cooling to room temperature, the solutions were transferred to a quartz cell or a CaF_2 cell and analyzed rapidly. For CD analyses at various concentrations (Figure 3c), solutions were obtained by dilution of the more concentrated one and this process was repeated several times until the concentration of **BTA P** in the mixture reached 0.09 mM.

Synthesis of BTA Eth, Figures S19-S23

Firstly, the amino ethers derived from (*S*)- and (*R*)-Leucinol were synthesized according to the following synthetic scheme**:**

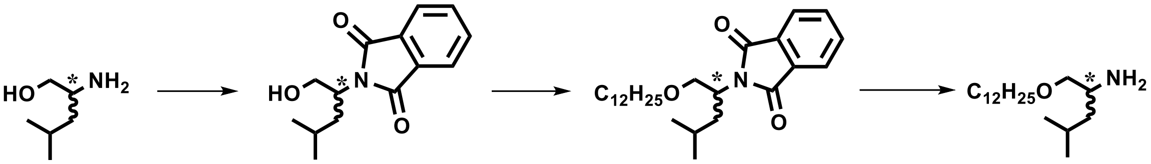

*= (***R***), (***R***)-1 *= (***R***), (***R***)-2 *= (***R***), (***R***)-3

*= (***S***), (***S***)-1 *= (***S***), (***S***)-2 *= (***S***), (***S***)-3

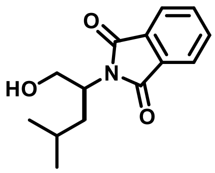

A solution of **(*S*)** or **(*R*)-Leucinol** (6.1 g, 52.0 mmol, 1.0 equiv.) in AcOEt (10 mL) was added to a suspension of phthalic anhydride (7.7 g, 52.0 mmol, 1.0 equiv.) in AcOEt (42 mL) and the mixture was refluxed overnight Then, the mixture was cooled to room temperature and water (30 mL) was added. Subsequently, the organic phase was washed thrice with water (30 mL) and twice with brine (20 mL) and evaporated under vacuum to yield a yellow oil. Pure **1** was obtained as a colourless oil after purification of the crude material by flash column chromatography over silica (heptane/AcOEt, 2:1). Yield: 80% for **(*R*)-1** and 75% for **(*S*)-1**. The ^{1}H NMR spectra of **(*R*)-1** and **(*S*)-1** are identical and match those reported.[14] **^{1}H NMR** ($CDCl_3$, 300 MHz) δ (ppm): 7.85–7.83 (m, AA'BB' pattern, 2H, CHarom.), 7.73–7.71 (m, AA'BB' pattern, 2H, CHarom.), 4.51–4.44 (m, 1H, CHN), 4.04 (dd, *J*= 11.7, 7.6 Hz, ABX pattern, 1H, OCH_2CH), 3.86 (dd, *J*= 11.7, 3.6 Hz, ABX pattern, 1H, OCH_2CH), 2.48 (br s, 1H, OH), 2.07–1.95 (m, 1H, C*H*(CH_3)$_2$), 1.66–1.48 (m, 2H, CHCH_2CH), 0.95 (d, *J*= 10.3 Hz, 3H, CH_3), 0.92 (d, *J*= 10.3 Hz, 3H, CH_3).

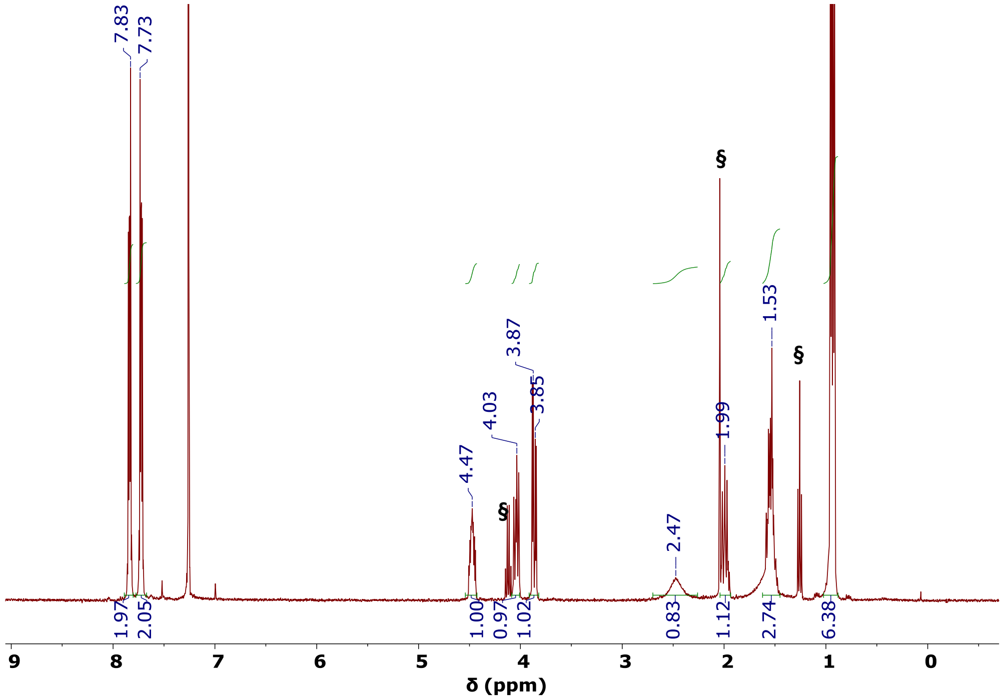

Figure S19. ^{1}H NMR of **(*R*)-1**. §= residual AcOEt.

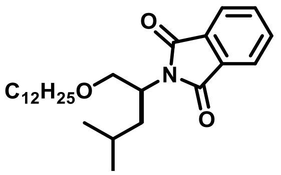

NaH (653 mg, 60% in oil, 16.3 mmol, 1.0 equiv.) was added in small portions at room temperature to a solution of **(*R*)-1** or **(*S*)-1** (4.0 g, 16.3 mmol, 1.0 equiv.) in dry THF (40 mL). C*areful: H_2 evolution was observed upon addition of NaH*. Subsequently, the reaction mixture was stirred gently for 15 min and then heated to 50 ºC for 2 hours where the colour of the mixture changed to yellow. The reaction was cooled down to room temperature and 1-bromododecane (16.1 mL, 65.2 mmol, 4.0 equiv.) in THF (40 mL) was added slowly to the mixture. The reaction was stirred at room temperature for additional 15 minutes and then refluxed overnight. The reaction mixture was cooled down to room temperature, the solvent was evaporated under vacuum and brine (30 mL) and AcOEt (50 mL) were added. The organic phase was extracted and the aqueous phase was washed twice with AcOEt (30 mL). The combined organic phases were collected, dried over anhydrous $MgSO_4$, filtered and evaporated under vacuum. The crude material was purified by flash column chromatography over silica (heptane to elute the 1-dodecene by-product, then heptane/AcOEt 1:3) to yield **2** and **2** as a yellow oil. Yield: 69% for **(*R*)-2** and 51% for **(*S*)-2**. The ^{1}H NMR spectra of **(*R*)-2** and **(*S*)-2** are identical. **^{1}H NMR** ($CDCl_3$, 300 MHz) δ (ppm): 7.85–7.79 (m, AA'BB' pattern, 2H, CHarom.), 7.72–7.66 (m, AA'BB' pattern, 2H, CHarom.), 4.61–4.51 (m, 1H, CHN), 3.93 (t, *J*= 9.8 Hz, 1H, OC*H*$_2$CH), 3.57 (dd, *J*= 10.0, 5.3 Hz, 1H, OC*H*$_2$CH), 3.50–3.43 (m, AA'XX' pattern, 1H, OC*H*$_2$CH$_2$), 3.34–3.27 (m, AA'XX' pattern, 1H, OC*H*$_2$CH$_2$), 2.08 (septet, *J*= 6.6 Hz, 1H, C*H*(CH$_3$)$_2$) 1.55–1.36 (m, 4H, CHC*H*$_2$CH + OCH$_2$C*H*$_2$), 1.32–1.04 (m, 18H, CH$_2$), 0.93 (d, *J*= 8.9 Hz, 3H, CHC*H*$_3$), 0.91 (d, *J*= 8.9 Hz, 3H, CHC*H*$_3$), 0.88 (t, *J*= 7.1 Hz, 3H, CH$_3$).

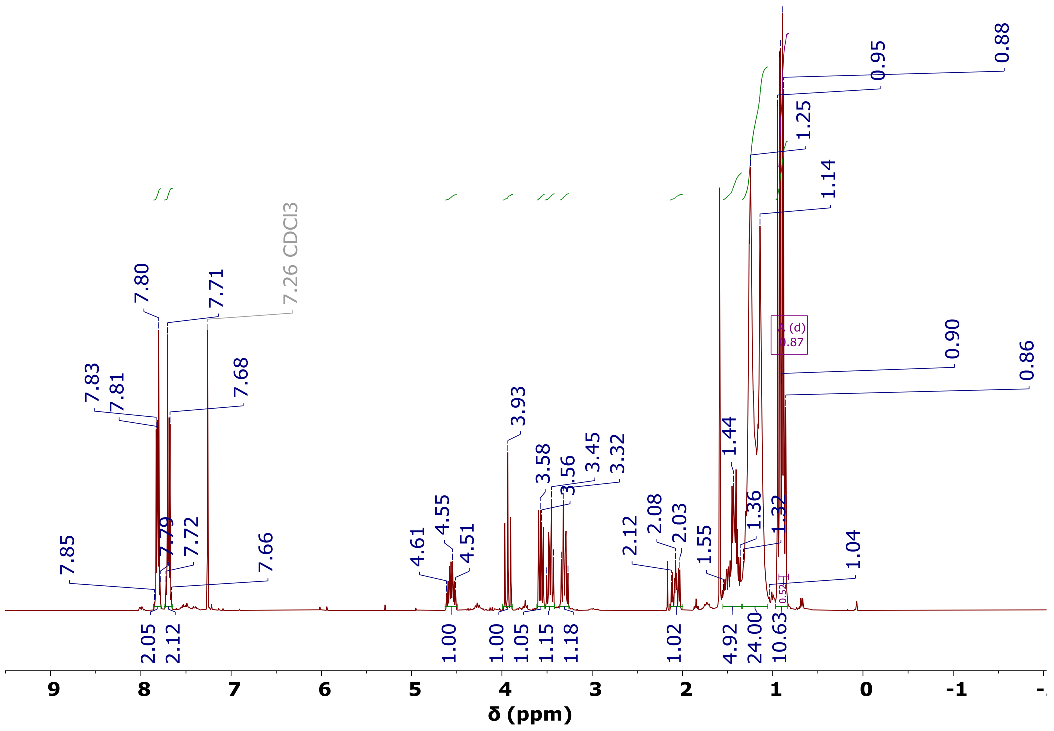

Figure S20. ^{1}H NMR of **(*R*)-2**.

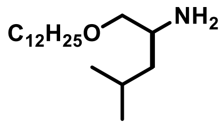

Hydrazine monohydrate (3.4 g, 60.2 mmol, 5.0 equiv.) was added to a solution of **(*R*)-2** or **(*S*)-2** in THF (50 mL) and the solution was refluxed overnight. The formed solid (phthalhydrazide) was discarded by filtration and AcOEt and brine was added to the solution. The organic phase was extracted, dried over $MgSO_4$ and evaporated under vacuum. The crude material was purified by flash column chromatography over silica (DCM/MeOH 9:1) to yield **3** as a pure yellowish oily solid. Yield: 90% for **(*R*)-3** and 85% for **(*S*)-3**. The ^{1}H NMR spectra of **(*R*)-3** and **(*S*)-3** are identical. **^{1}H NMR** ($CDCl_3$, 300 MHz) δ (ppm): 3.48–3.43 (m, 3H, CHN + OCH_2CH+ OCH_2CH$_2$), 3.10 (t, *J*= 8.2 Hz,1H, OCH_2CH), 3.06–2.97 (m, 1H, OCH_2CH$_2$), 1.72 (septet, *J*= 6.6 Hz, 1H, C*H*(CH$_3$)$_2$), 1.59–1.51 (m, 4H, CHCH_2CH + OCH$_2$CH_2) 1.35–1.12 (m, 18H, CH$_2$), 1.19–1.14 (m, 2H, NH$_2$), 0.92 (d, *J*= 8.9 Hz, 3H, CHCH_3), 0.89 (d, *J*= 8.9 Hz, 3H, CHCH_3), 0.87 (t, *J*= 7.1 Hz, 3H, CH$_3$).

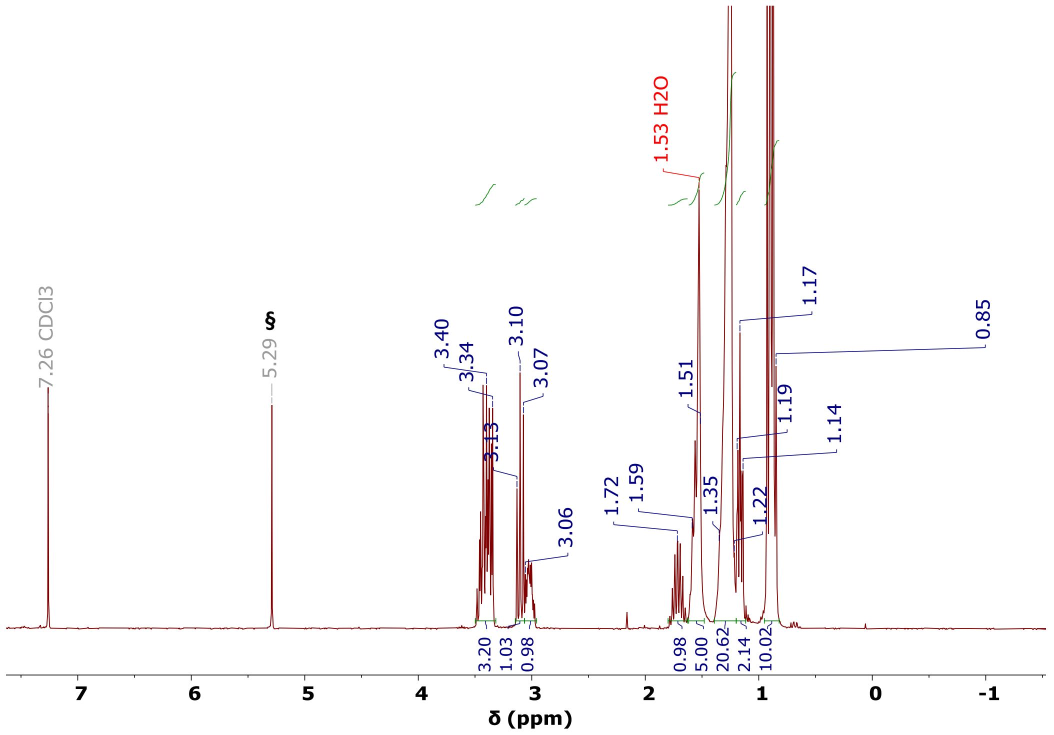

Figure S21. ^{1}H NMR of **(*R*)-3**. §= residual DCM.

Synthesis of BTA (*R*)-Eth and BTA (*S*)-Eth:

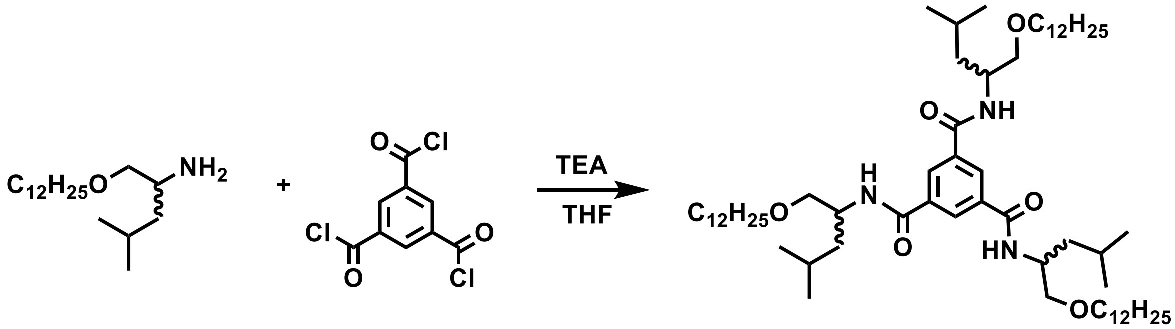

*= (*R*), (*R*)-3
*= (*S*), (*S*)-3

*= (*R*), BTA (*R*)-Eth
*= (*S*), BTA (*S*)-Eth

In a dry round-bottom flask under argon atmosphere and at room temperature, benzene-1,3,5-tricarbonyl trichloride (141 mg, 0.53 mmol, 1.0 equiv.) and **(*R*)-3** or **(*S*)-3** (500 mg, 1.75 mmol, 3.3 equiv.) were dissolved in dry THF (5 mL). Dry NEt_3 (0.27 mL, 1.9 mmol, 3.6 equiv.) was then added dropwise and the reaction was stirred overnight. The mixture was then evaporated and AcOEt (20 mL) was added to the crude. The organic phase was washed with aqueous HCl (0.1 M, 10 mL), water and brine, dried over $MgSO_4$, filtered and evaporated under vacuum. The crude material was recrystallized from acetonitrile yielding **BTA Eth** as a colorless solid. Yields: 70% for **BTA (*R*)-Eth** and 75% for **BTA (*S*)-Eth**. The ^{1}H NMR spectra of **BTA (*R*)-Eth** and **BTA (*S*)-Eth** are identical. Overall yield for the four steps: 35% for **BTA (*R*)-Eth** and 24% for **BTA (*S*)-Eth.** **^{1}H NMR** (acetone-*d*6, 400 MHz) δ (ppm): 8.41 (s, 3H, CHarom.), 7.77 (d, *J*= 8.7 Hz, 3H, NH), 4.46–4.37 (m, 3H, CHN), 3.55–3.40 (m, 12H, OCH_2CH+ OCH_2CH_2), 1.81–1.69 (m, 3H, $CH(CH_3)_2$), 1.65–1.43 (m, 12H, $CHCH_2CH$ + OCH_2CH_2), 1.40–1.20 (m, 54H, CH_2), 0.95 (d, *J*= 8.9 Hz, 9H, $CHCH_3$), 0.93 (d, *J*= 8.9 Hz, 9H, $CHCH_3$), 0.87 (t, *J*= 7.1 Hz, 9H, CH_3). **^{13}C JMOD NMR** (acetone-*d*6, 101 MHz) δ (ppm): 166.11 (CO), 136.40 (Carom.), 129.18 (CHarom.), 73.89 (OCH_2CH), 71.63 (OCH_2CH_2), 48.58 (CHN), 41.54 (CH_2), 32.64 (CH_2), 30.46 (CH_2), 30.39 (CH_2), 30.36 (CH_2), 30.20 (CH_2), 30.08 (CH_2), 26.94 (CH_2), 25.57 ($CHCH_3$), 23.77 ($CHCH_3$), 23.33 (CH_2), 22.34 ($CHCH_3$), 14.37 (CH_3). HRMS (ESI, *m/z*): calculated for $C_{63}H_{117}N_3O_6H$, $[M+H]^+$: 1011.8942, found 1011.7892.

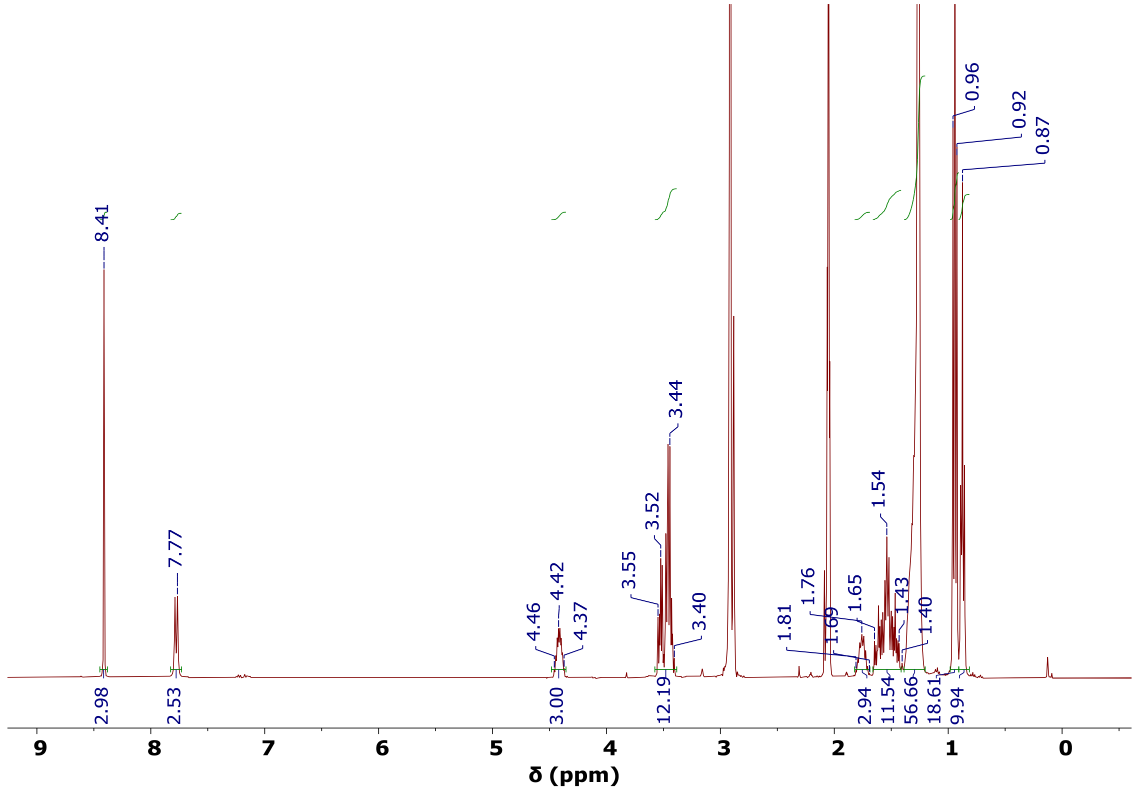

Figure S22. ^{1}H NMR of **BTA (*R*)-Eth**. Signals at *ca.* 2.9 ppm are water and HDO.

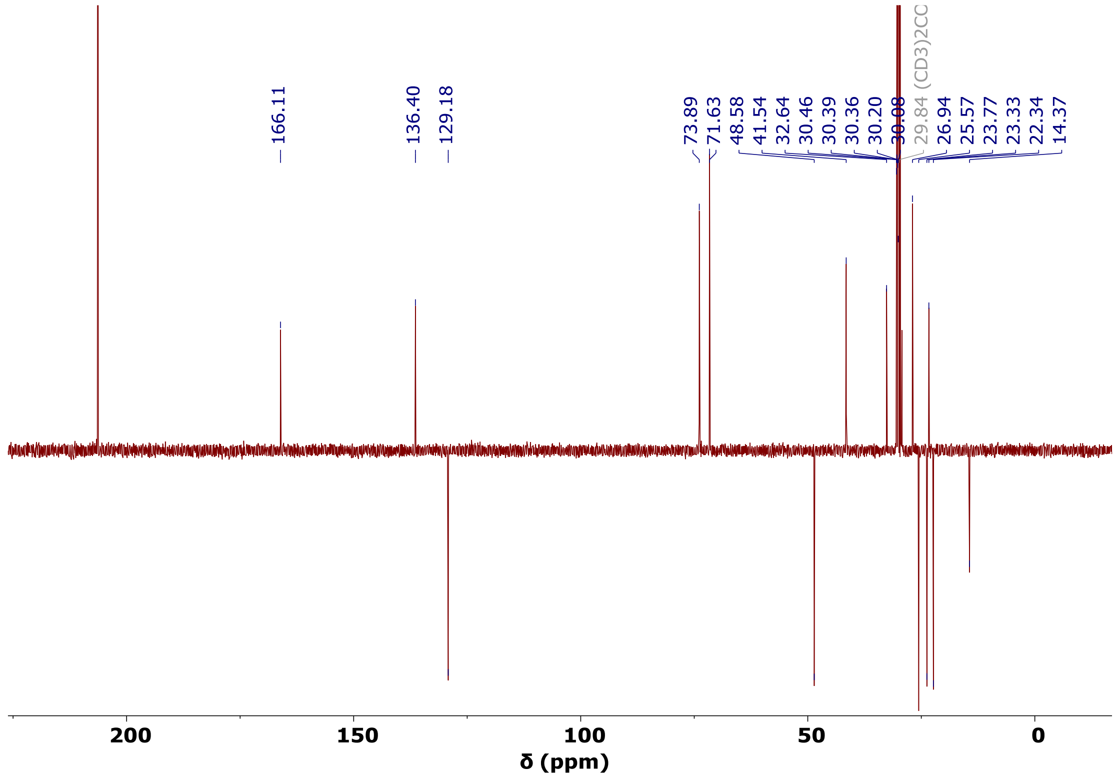

Figure S23. ^{13}C JMOD-NMR of **BTA (*R*)-Eth.**

Optical purity of BTA (*S*)-Eth and BTA (*R*)-Eth, Figure S24

Result: **BTA (*S*)-Eth** (>99% ee, >93% de).

Result: **BTA (*R*)-Eth** (>99% ee, >99% de).

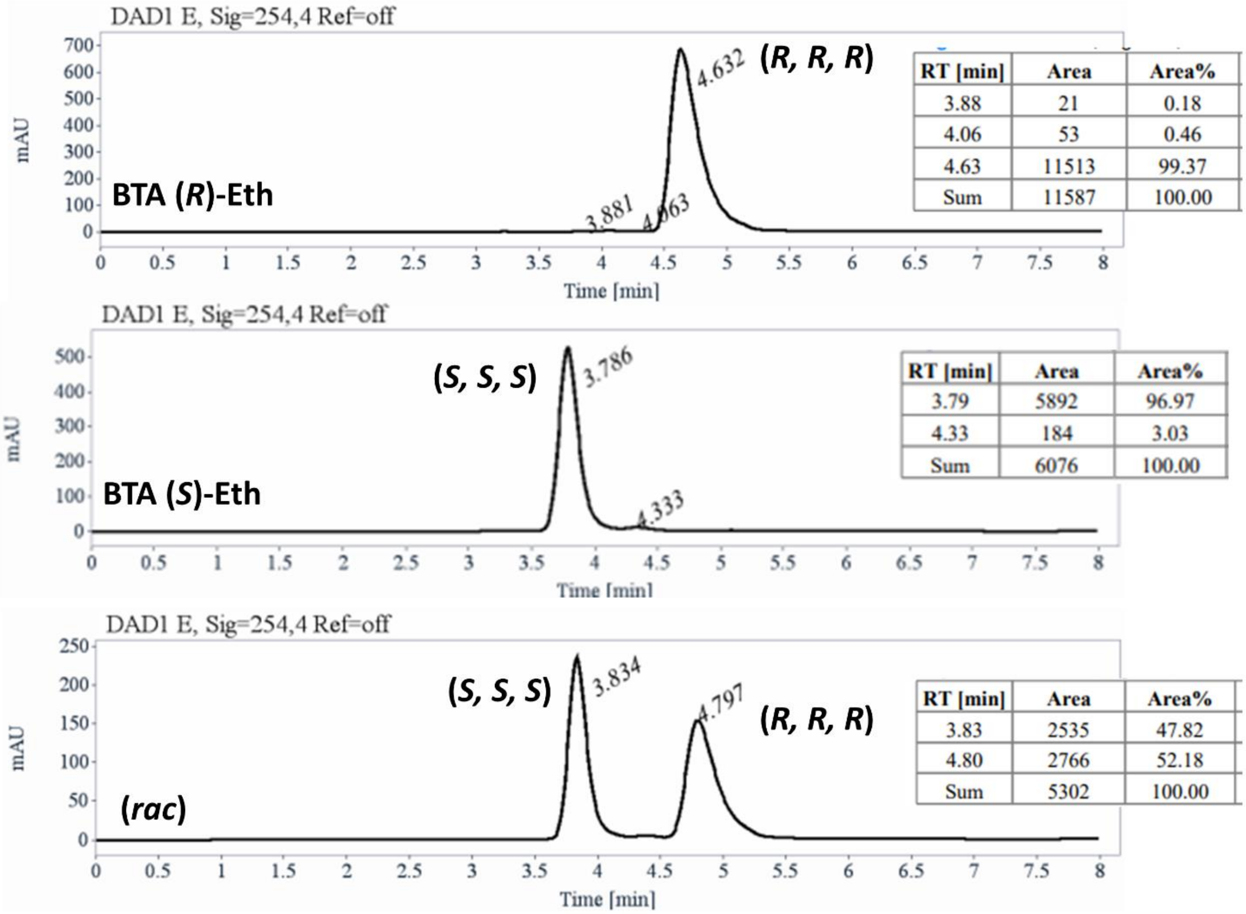

RT [min]	Area	Area%
3.88	21	0.18
4.06	53	0.46
4.63	11513	99.37
Sum	11587	100.00

RT [min]	Area	Area%
3.79	5892	96.97
4.33	184	3.03
Sum	6076	100.00

RT [min]	Area	Area%
3.83	2535	47.82
4.80	2766	52.18
Sum	5302	100.00

Figure S24. Chiral HPLC analyses **of BTA (*S*)-Eth,** of **BTA (*R*)-Eth** and of their 1:1 mixture.

Selected chiral GC analyses, Figures S25-S37

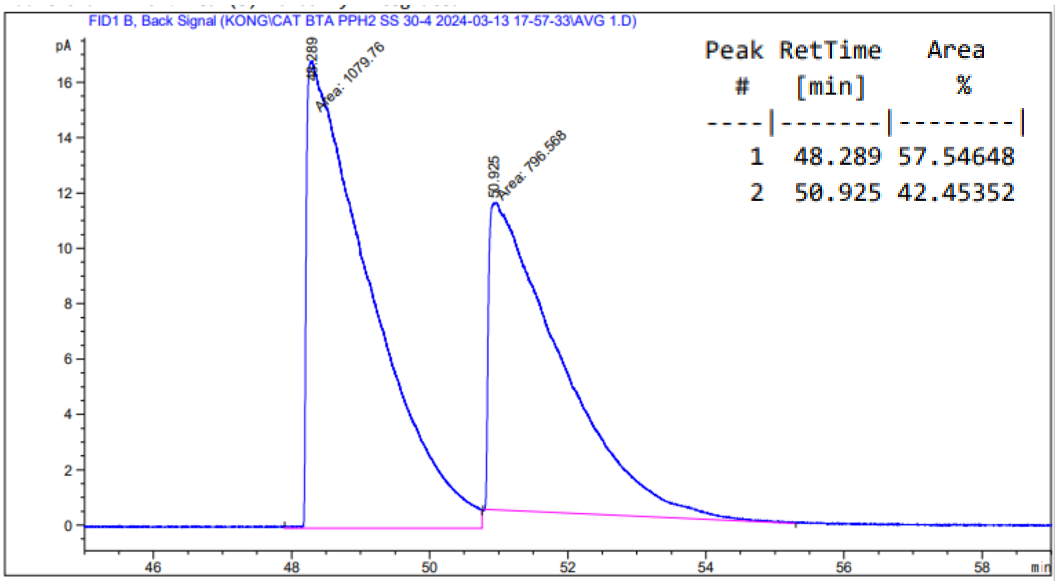

Figure S25. Chiral GC analysis of entry 2, Table S5. Composition: **BTA P**/[Cu]= 4, **BTA P**= 0.58 mM, **BTA (*S*)-Est** (fs_0 = 5%). ─15% *e.e.*

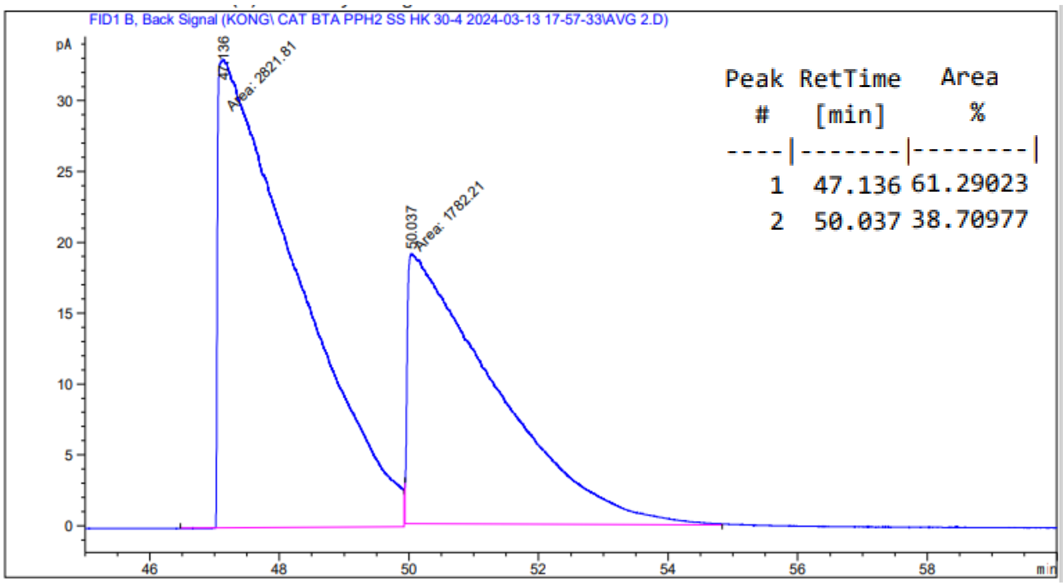

Figure S26. Chiral GC analysis of entry 3, Table S5. Composition: **BTA P**/[Cu]= 4, **BTA P**= 0.58 mM, **BTA (*S*)-Est** (fs_0 = 10%). ─22% *e.e.*

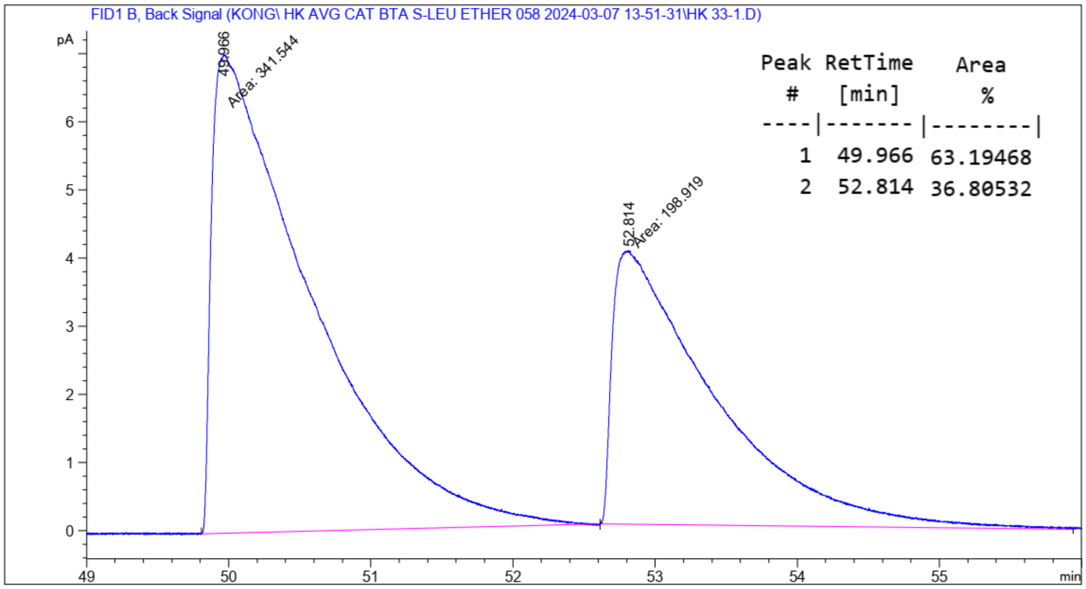

Figure S27. Chiral GC analysis of entry 4, Table S5. Composition: **BTA P**/[Cu]= 4, **BTA P**= 0.58 mM, **BTA (*S*)-Est** (fs_0 = 20%). ─26% *e.e.*

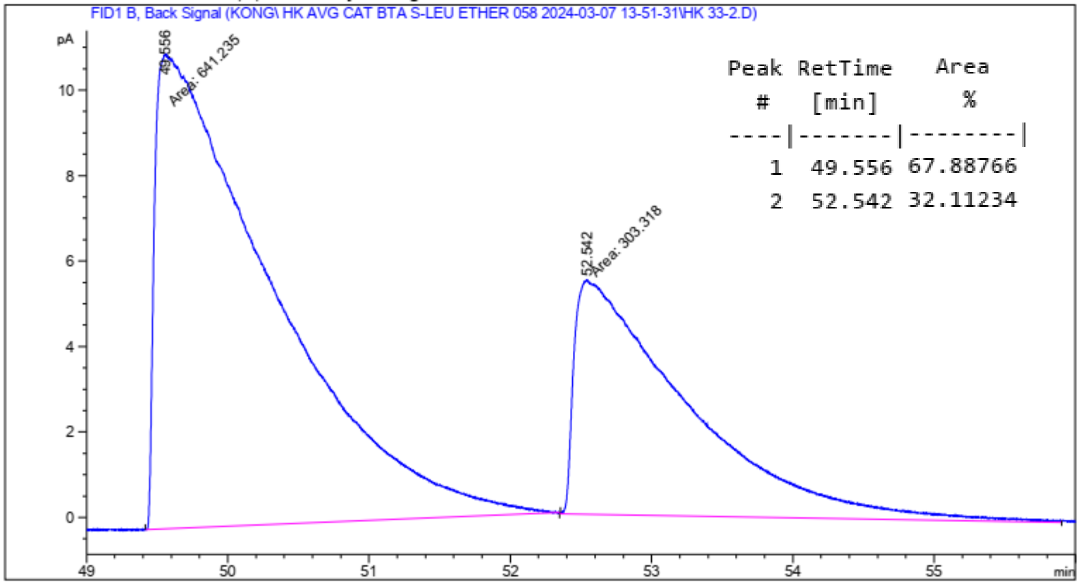

Figure S28. Chiral GC analysis of entry 5, Table S5. Composition: **BTA P**/[Cu]= 4, **BTA P**= 0.58 mM, **BTA (*S*)-Est** (fs_0 = 40%). ─36% *e.e.*

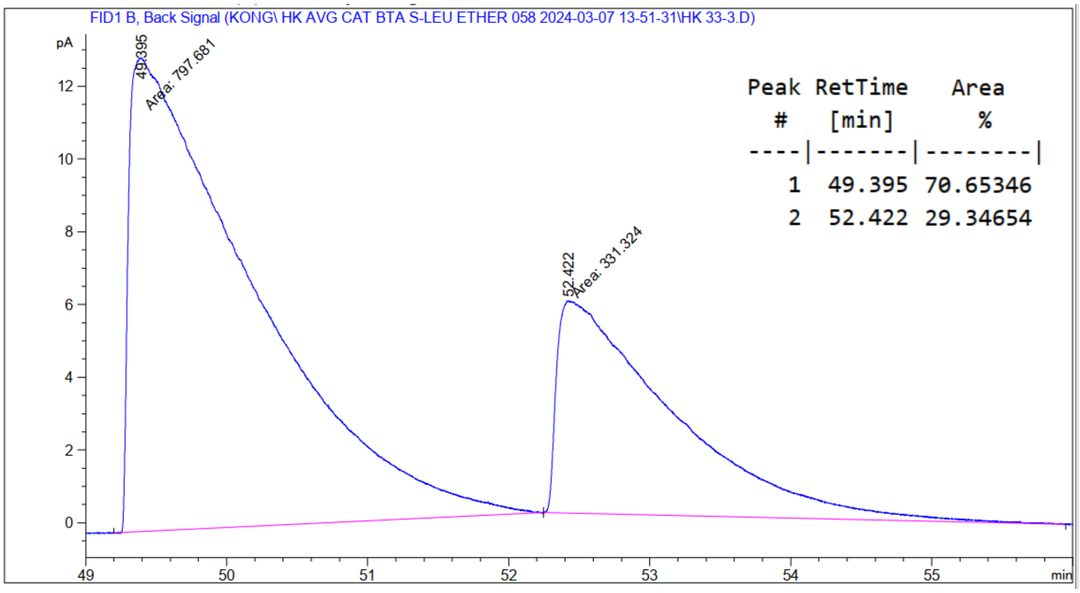

Figure S29. Chiral GC analysis of entry 6, Table S5. Composition: **BTA P**/[Cu]= 4, **BTA P**= 0.58 mM, **BTA (*S*)-Est** (fs_0 = 50%). —41% *e.e.*

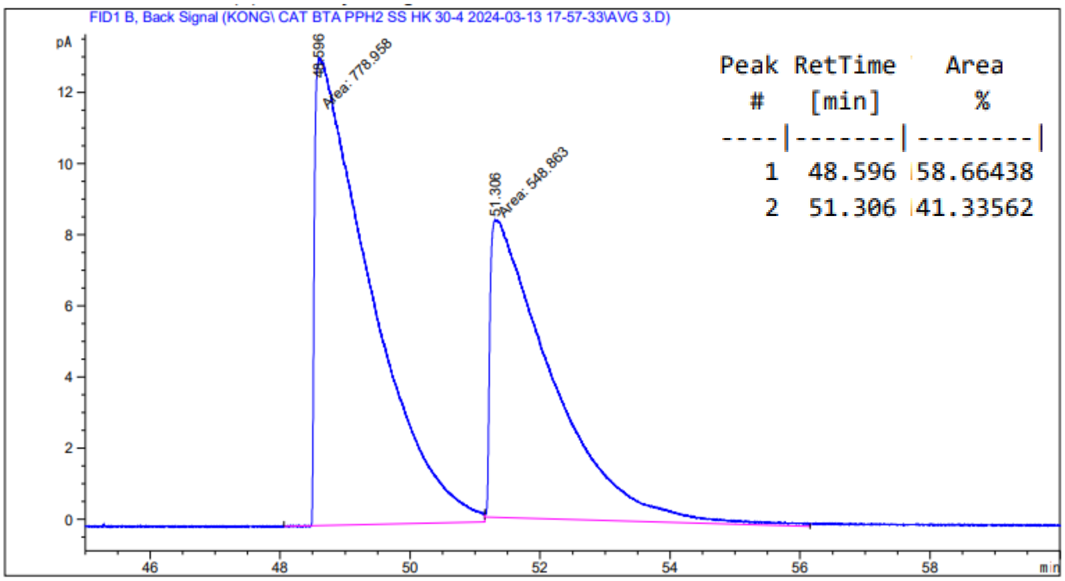

Figure S30. Chiral GC analysis of entry 3, Table S5. Composition: **BTA P**/[Cu]= 4, **BTA P**= 0.58 mM, **BTA (*S*)-Eth** (fs_0 = 5%). —17% *e.e.*

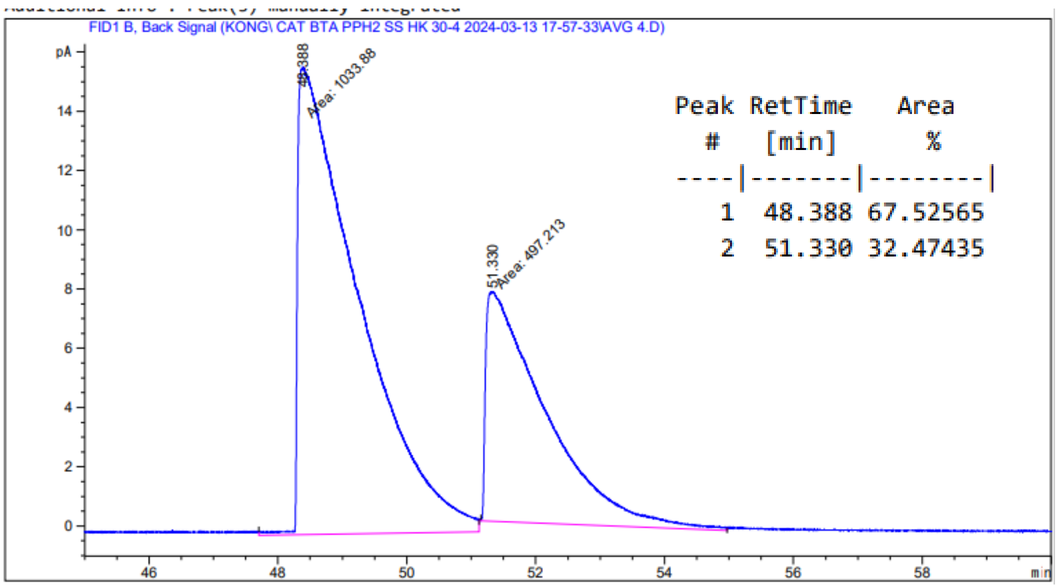

Figure S31. Chiral GC analysis of entry 4, Table S6. Composition: **BTA P**/[Cu]= 4, **BTA P**= 0.58 mM, **BTA (*S*)-Eth** (fs_0 = 10%). −35% *e.e.*

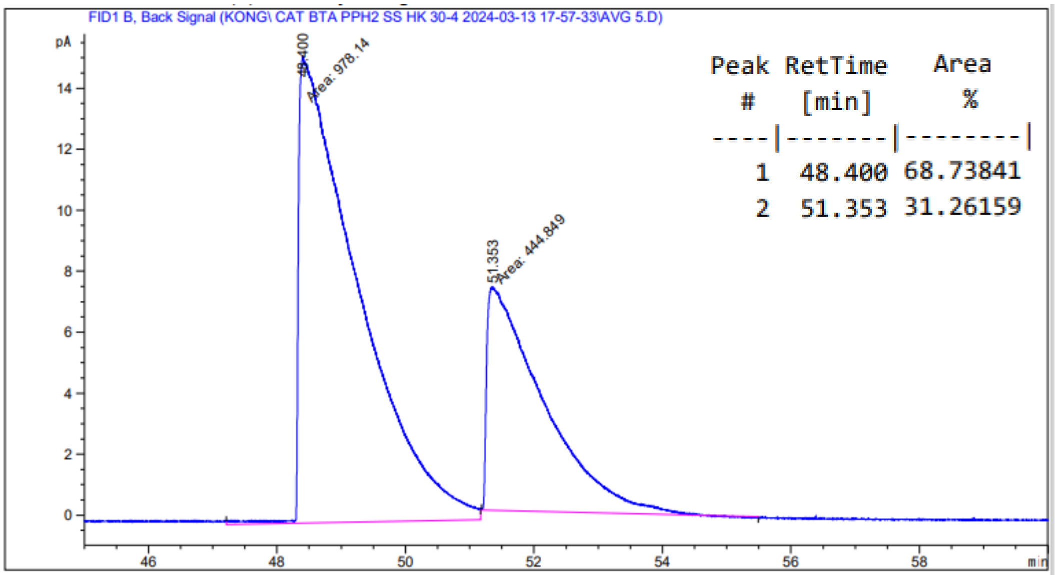

Figure S32. Chiral GC analysis of entry 4, Table S6. Composition: **BTA P**/[Cu]= 4, **BTA P**= 0.58 mM, **BTA (*S*)-Eth** (fs_0 = 15%). −37% *e.e.*

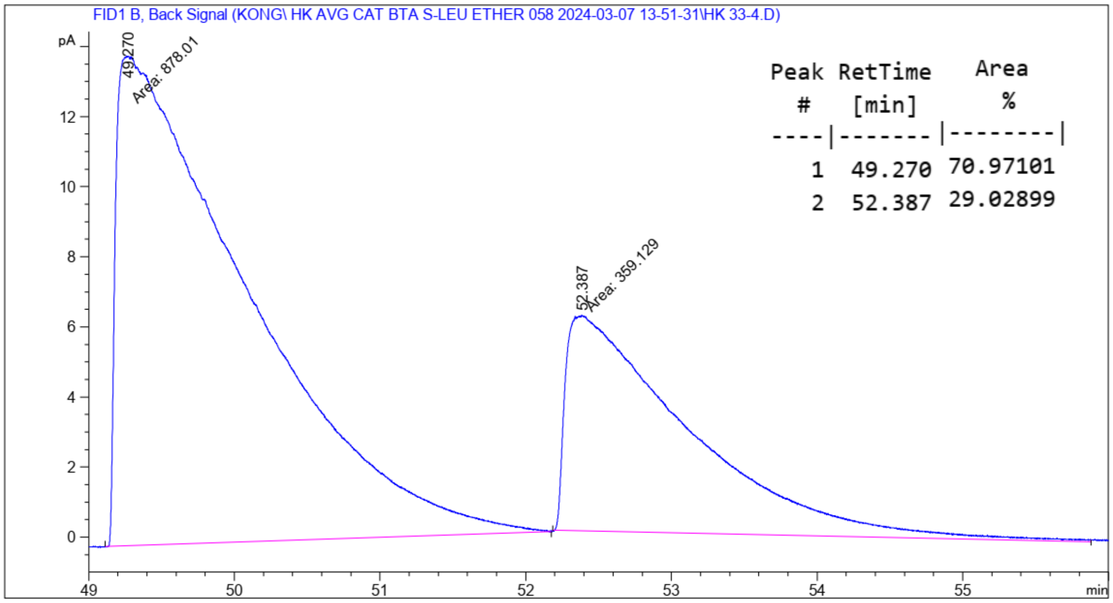

Figure S33. Chiral GC analysis of entry 5, Table S6. Composition: **BTA P**/[Cu]= 4, **BTA P**= 0.58 mM, **BTA (*S*)-Eth** (fs_0 = 20%). −42% *e.e.*

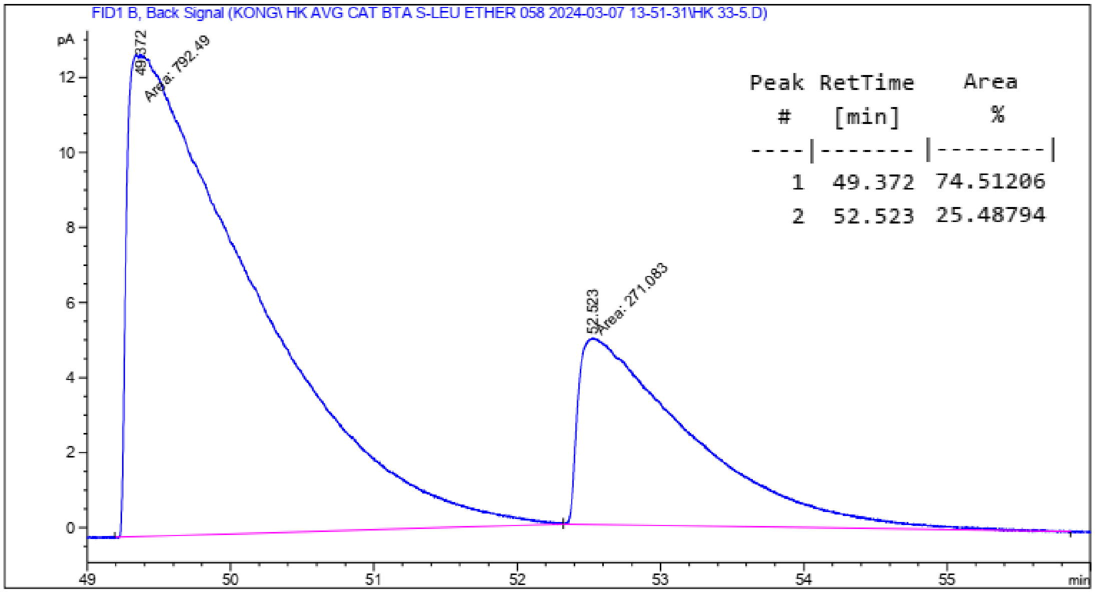

Figure S34. Chiral GC analysis of entry 6, Table S6. Composition: **BTA P**/[Cu]= 4, **BTA P**= 0.58 mM, **BTA (*S*)-Eth** (fs_0 = 40%). −49% *e.e.*

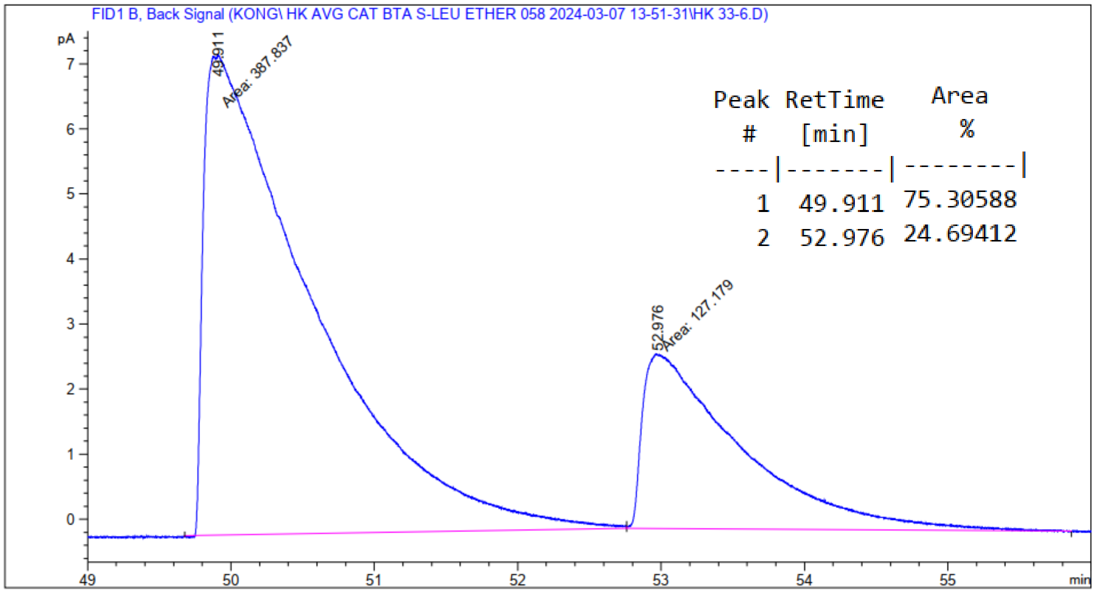

Figure S35. Chiral GC analysis of entry 7, Table S6. Composition: **BTA P**/[Cu] = 4, **BTA P**= 0.58 mM, **BTA (*S*)-Eth** (fs_0 = 50%). ─51% *e.e.*

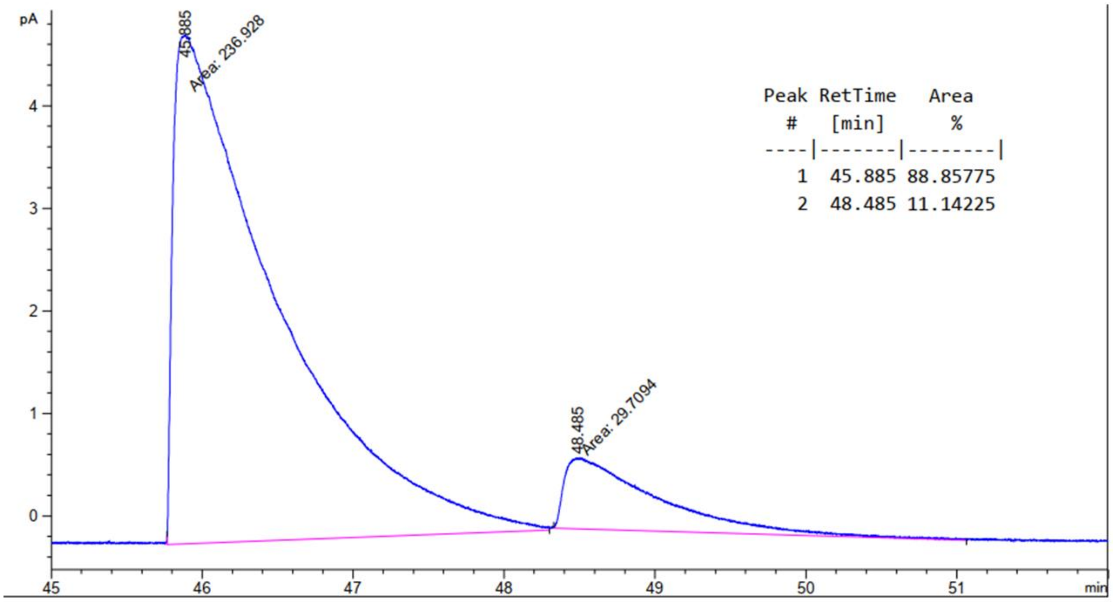

Figure S36. Chiral GC analysis of entry 3, Figure S16. Composition: **BTA P^{Me}**/[Cu] = 4, **BTA P^{Me}**= 0.58 mM, **BTA (*S*)-Eth** (fs_0 = 50%). ─78% *e.e.*

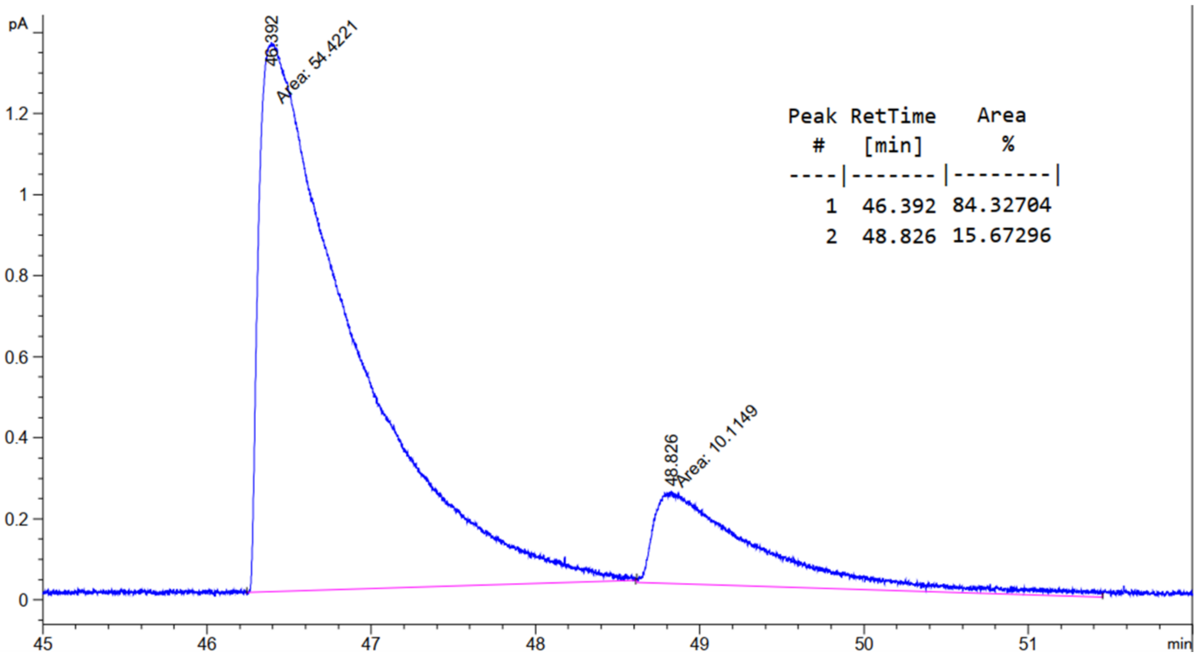

Figure S37. Chiral GC analysis of entry 4, Figure S16. Composition: **BTA P^{Me}**/[Cu] = 4, **BTA P^{Me}**= 0.58 mM, **BTA (*S*)-Est** (fs_0 = 50%). ─69% *e.e.*

References

[1] A. Desmarchelier, M. Raynal, P. Brocorens, N. Vanthuyne, L. Bouteiller, *Chem. Commun.* **2015**, *51*, 7397–7400.

[2] A. Desmarchelier, B. G. Alvarenga, X. Caumes, L. Dubreucq, C. Troufflard, M. Tessier, N. Vanthuyne, J. Idé, T. Maistriaux, D. Beljonne, P. Brocorens, R. Lazzaroni, M. Raynal, L. Bouteiller, *Soft Matter* **2016**, *12*, 7824–7838.

[3] X. Caumes, A. Baldi, G. Gontard, P. Brocorens, R. Lazzaroni, N. Vanthuyne, C. Troufflard, M. Raynal, L. Bouteiller, *Chem. Commun.* **2016**, *52*, 13369–13372.

[4] J. G. De La Torre, M. C. L. Martinez, M. M. Tirado, *Biopolymers* **1984**, *23*, 611–615.

[5] P. Evenou, J. Rossignol, G. Pembouong, A. Gothland, D. Colesnic, R. Barbeyron, S. Rudiuk, A.-G. Marcelin, M. Ménand, D. Baigl, V. Calvez, L. Bouteiller, M. Sollogoub, *Angew. Chem. Int. Ed.* **2018**, *57*, 7753–7758.

[6] G. Vantomme, G. M. ter Huurne, C. Kulkarni, H. M. M. ten Eikelder, A. J. Markvoort, A. R. A. Palmans, E. W. Meijer, *J. Am. Chem. Soc.* **2019**, *141*, 18278–18285.

[7] M. A. Martínez-Aguirre, Y. Li, N. Vanthuyne, L. Bouteiller, M. Raynal, *Angew. Chem. Int. Ed.* **2021**, *60*, 4183–4191.

[8] A. Hammoud, Y. Li, M. A. Martínez-Aguirre, H. Kong, L. Dubreucq, C. Troufflard, L. Bouteiller, M. Raynal, *Chem. – Eur. J.* **2023**, *29*, e202300189.

[9] Y. Li, A. Hammoud, L. Bouteiller, M. Raynal, *J. Am. Chem. Soc.* **2020**, *142*, 5676–5688.

[10] S. Allenmark, *Chirality* **2003**, *15*, 409–422.

[11] J. M. Zimbron, X. Caumes, Y. Li, C. M. Thomas, M. Raynal, L. Bouteiller, *Angew. Chem. Int. Ed.* **2017**, *56*, 14016–14019.

[12] A. Desmarchelier, X. Caumes, M. Raynal, A. Vidal-Ferran, P. W. N. M. van Leeuwen, L. Bouteiller, *J. Am. Chem. Soc.* **2016**, *138*, 4908–4916.

[13] P. J. M. Stals, M. M. J. Smulders, R. Martín-Rapún, A. R. A. Palmans, E. W. Meijer, *Chem. – Eur. J.* **2009**, *15*, 2071–2080.

[14] M. F. Lindberg, E. Deau, F. Miege, M. Greverie, D. Roche, N. George, P. George, L. Merlet, J. Gavard, S. J. T. Brugman, E. Aret, P. Tinnemans, R. de Gelder, J. Sadownik, E. Verhofstad, D. Sleegers, S. Santangelo, J. Dairou, Á. Fernandez-Blanco, M. Dierssen, A. Krämer, S. Knapp, L. Meijer, *J. Med. Chem.* **2023**, *66*, 15648–15670.
